# Synergistic anticancer effect of *Pistacia lentiscus* essential oils and 5-Fluorouracil co-loaded onto biodegradable nanofibers against melanoma and breast cancer

**DOI:** 10.1186/s11671-024-03962-5

**Published:** 2024-02-14

**Authors:** Obaydah Abd Alkader Alabrahim, Hassan Mohamed El-Said Azzazy

**Affiliations:** 1 Department of Chemistry, School of Sciences & Engineering, The American University in Cairo, AUC Avenue, SSE # 1184, P.O. Box 74, New Cairo, 11835 Egypt; 2 Department of Nanobiophotonics, Leibniz Institute of Photonic Technology, Albert Einstein Str. 9, Jena, Germany

**Keywords:** Drug delivery, Nanofibers, Chemotherapeutics, 5-Fluorouracil, Natural extracts, Essential oils, *Pistacia lentiscus*, Synergistic anticancer activity, Breast cancer, Melanoma

## Abstract

Chemoresistance and severe toxicities represent major drawbacks of chemotherapy. Natural extracts, including the essential oils of *Pistacia lentiscus* (PLEO), exhibit substantial anticancer and anti-inflammatory activities where different cancers are reported to dramatically recess following targeting with PLEO. PLEO has promising antimicrobial, anticancer, and anti-inflammatory properties. However, the therapeutic properties of PLEO are restricted by limited stability, bioavailability, and targeting ability. PLEO nanoformulation can maximize their physicochemical and therapeutic properties, overcoming their shortcomings. Hence, PLEO was extracted and its chemical composition was determined by GC–MS. PLEO and 5-Fluorouracil (5FU) were electrospun into poly-ε-caprolactone nanofibers (PCL-NFs), of 290.71 nm to 680.95 nm diameter, to investigate their anticancer and potential synergistic activities against triple-negative breast cancer cells (MDA-MB-231), human adenocarcinoma breast cancer cells (MCF-7), and human skin melanoma cell line (A375). The prepared nanofibers (NFs) showed enhanced thermal stability and remarkable physical integrity and tensile strength. Biodegradability studies showed prolonged stability over 42 days, supporting the NFs use as a localized therapy of breast tissues (postmastectomy) or melanoma. Release studies revealed sustainable release behaviors over 168 h, with higher released amounts of 5FU and PLEO at pH 5.4, indicating higher targeting abilities towards cancer tissues. NFs loaded with PLEO showed strong antioxidant properties. Finally, NFs loaded with either PLEO or 5FU depicted greater anticancer activities compared to free compounds. The highest anticancer activities were observed with NFs co-loaded with PLEO and 5FU. The developed 5FU-PLEO-PCL-NFs hold potential as a local treatment of breast cancer tissues (post-mastectomy) and melanoma to minimize their  possible recurrence.

**Graphical Abstract:**

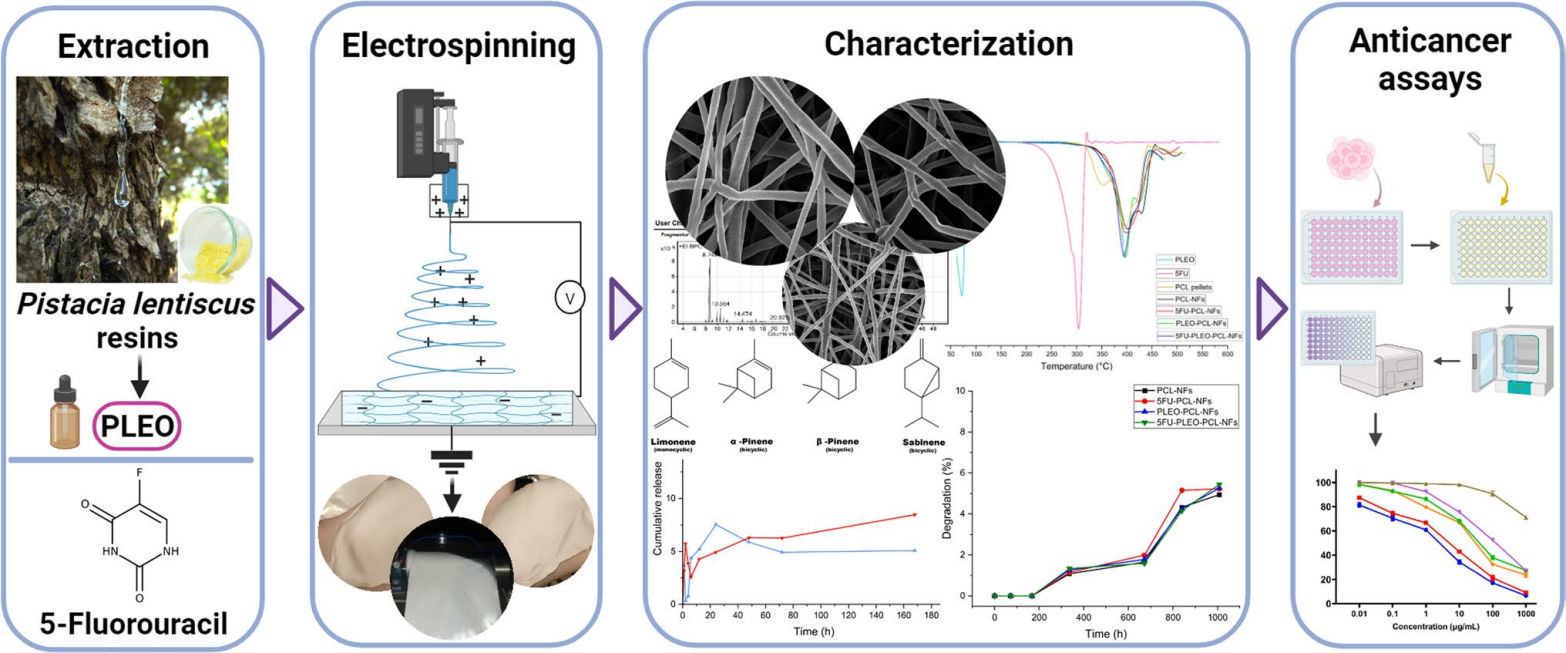

**Supplementary Information:**

The online version contains supplementary material available at 10.1186/s11671-024-03962-5.

## Introduction

Cancer is still considered the prime cause of death worldwide, ranking the first or second cause of death among 112 countries in 2019, according to the World Health Organization (WHO) [1, 2]. Recent reports highlighted higher mortality rates of cancer among various countries, in some cases surpassing those of coronary heart diseases [1]. Apparently, there is a rapid growth in the mortality and incidence rates of cancer, a fact that is further supported by the latest statistical report and study published by the International Agency for Research on Cancer, the Global Cancer Observatory (GLOBOCAN), 2020 [3]. The GLOBOCAN reported that 19.3 million patients were diagnosed with cancer, whereas 10 million patients died due to cancer in 2020 worldwide [3]. More importantly, female breast cancer had the most significant number of diagnosed cases, exceeding lung cancer for the first time, with 2.3 million (out of 9.3 million) newly diagnosed cases with female breast cancer (11.7%), followed by lung cancer (11.4%) [3]. Also, female breast cancer came in fifth (6.9%) in the number of deaths. On the other hand, melanoma was responsible for the most skin cancer-related deaths, with 324,635 (1.7%) newly diagnosed cases and 57,034 (0.6%) deaths [3].

Different criteria were utilized to determine the most effective and personalized treatment for breast and melanoma cancers. The general treatment approaches include surgery, radiotherapy, and chemotherapy [4–8]. Several shortcomings have been linked with radiation therapy, including cardiotoxicity and radiation resistance [9–11]. Additionally, various chemotherapeutic groups have been utilized for breast cancer treatment, such as alkylating agents (e.g., cyclophosphamide), taxanes (e.g., paclitaxel and docetaxel), and antimetabolites (e.g., 5-Fluorouracil, 5FU, and methotrexate) [12, 13]. Severe and chronic toxicities, in addition to chemoresistance, represent the major drawbacks of chemotherapy in treating all cancers, which negatively impacts all organ systems [12, 14–16]. Early toxicities commonly involve alopecia, musculoskeletal disorders, peripheral neuropathy, fatigue, cytopenia, neurocognitive malfunctions, etc. In addition to tumors relapsing and recurrence, chronic toxicities involve psychological disorders, cardiomyopathy, sterility, myelosuppression, neurotoxicity, osteoarthritis, alopecia, gastrointestinal disorders, peripheral neuropathies, thromboembolisms, etc [12, 14–16].

The higher incidence of metastases and recurrence (30% of the early-stage cases) [7] and the drawbacks of the current approaches employed for cancer management have warranted the urgent need to design alternative treatment approaches and strategies [17–19]. Recent reports have shown promising findings of natural extracts and essential oils (EOs) encapsulated into nanofibers for various cancer treatments [20–27]. Biodegradable and biocompatible nanofibers loaded with natural extracts, which contain different bioactive metabolites with direct and indirect anticancer and cytotoxic activities, represent a promising approach for treating different cancers [28].

Many natural compounds derived from various plant extracts have exerted potent anticancer activities. Hence, natural compounds have been widely utilized by 80% of the population worldwide [29]. Three thousand plant species were reported for cancer therapy in modern medicine [30]. Such compounds can target cancerous cells and induce their death via several pathways, including apoptosis and autophagic pathways activation [29].

EOs refer to a large group of secondary metabolites extracted from higher plants, with unique characteristics of low molecular weight, pungent odor, aromatic, and volatile nature. Such characteristics are derived from the natural multiple ingredients of EOs which primarily include volatile terpenes and hydrocarbons [31]. EOs have shown a wide variety of beneficial properties for pharmaceutical and biomedical applications, owing to their outstanding medicinal (*e.g.*, spasmolytic, sedative, local anesthetic, anti-inflammatory, analgesic, anticancer) and antiseptic (*e.g.*, antifungal, antibacterial, antiviral) activities [32]. More than three thousand EOs have been identified, and only three hundred of these EOs have been established for their well-commercial appeal, particularly for cosmetic, food, perfume, agronomic, pharmaceutical, and sanitary industries [31].

Various tumorous tissues have shown great recession after being targeted and treated with EOs of different plants. Several malignancies were targeted by EOs, such as leukemia, hepatoma, breast tumors, gastric malignancies, glioma, and pulmonary and colorectal cancers. Therefore, EOs have been established with great potential for various preventive and therapeutic strategies amongst different tumors. [33–35]

Although EOs have been established for various and outstanding biological applications, several limitations still hinder the utmost exploitation of their properties and activities, particularly for clinical applications. Such limitations include poor solubility and bioavailability (due to their hydrophobic nature), high volatility, chemical instability (at high temperatures and oxygen), and humidity and light sensitivity [36]. Therefore, it has been essential to explore and discover new strategies for EOs' delivery and application, particularly for cancer targeting and therapy, to achieve maximum biological benefits. [36]

Nanoformulated drug delivery systems have shown superior characteristics. These include higher sustainable drug release, cargo protection, outstanding drug permeability against tumorous cells and tissues, enhanced drug bioavailability and solubility, simultaneous drug loading ability, amenability for functionalization, and improved therapeutic efficacy [37–41]. Such characteristics have facilitated delivery of several drugs, small molecules, biological entities, and natural extracts while overcoming their limitations [37–41]. Eventually, in addition to the auspicious intrinsic therapeutic properties of some nanomaterials, the nanoscale features have provided promising solutions. These have further been well-established for drug delivery while loading current applied therapies to augment their overall therapeutic efficiency particularly for cancer targeting and treatment. [37]

Moreover, nanoformulations can eradicate various tumors and overcome their resistance mechanisms via several approaches while enhancing the triggering, accumulation, specificity, and localization abilities of their loaded drug(s) [42, 43]. Indeed, multiple nanocarriers have exploited the poor lymphatic drainage and leaky blood vessels surrounding tumor tissues to deliver and bypass the tumor endothelium and passively accumulate inside [44]. Additionally, other nanomaterials have established better prognosis and early detection and monitoring of several cancers [45, 46]. The outstanding chemical and physical properties of nanomaterials have facilitated their effective exploitation for complex and yet highly sensitive diagnostic tests and imaging techniques [45, 46]. This has further allowed for improving tumors surveillance, surgical guidance in tumors resection, and treatments monitoring [47, 48]. Hence, nanomaterials have been extensively utilized for managing various cancers in terms of treatment, imaging, diagnostic, and overall therapeutic applications [45–48].

Lipid-based nanoformulations (*e.g.*, liposomes) predominate the clinically approved nanomedicines for cancer-targeting applications [49]. However, other drug conjugates and nanoformulations have also been approved such as polymeric, including polymeric nanoparticles and nanofibers, protein, and antibody-based drug conjugates [40, 49]. Such nano-medicinal formulations have mainly focused on enhancing the pharmacodynamic/kinetic properties, passive targeting, and overall therapeutic efficacy of free drugs [49–52]. Hence, several nanosystems have been exploited for the delivery of different drugs and bioactive compounds for tumor targeting, including polymeric, lipid, and inorganic nanomaterials [39, 41, 49–52].

Nanofibers signify a group of nanomaterials produced commonly via electrospinning, with a diameter range of tens to hundreds of nanometers [53]. Several other methods have been investigated for the production of nanofibers, including template synthesis, phase separation, and self-assembly [54–56]. Nevertheless, electrospinning is considered the most utilized technique to produce nanofibers with exceptional properties for encapsulating and loading natural extracts, essential oils, chemotherapeutics, and other drugs. This is due to the cost-effective, simple, rapid, and continuous properties which characterize the electrospinning process [57]. Moreover, electrospun nanofibers can be prepared using various synthetic, natural, and hybrid polymers to develop fibers with diameters of a few nanometers and a very large surface area. Additionally, electrospun nanofibers have been established with auspicious mechanical strength properties, ease of functionalization, large interstitial spaces, excellent chemical and physical properties, and scaling-up feasibility [20, 53].

The encapsulation of natural extracts, EOs in particular, into nanofibers has motivated the development of novel treatment strategies including targeted delivery of drugs, scaffolds for tissue engineering, and wound healing dressings. Furthermore, encapsulating EOs into nanofibers could increase their stability, bioavailability, sustainability, targeting ability, and overall therapeutic efficacy while reducing their side effects and limitations mentioned earlier [20, 21]. For instance, curcumin was loaded onto PCL-PEG-PCL and PLGA polymeric nanofibers, showing greater anticancer activities against glioma and epidermoid carcinoma, respectively. [22, 23] The encapsulation of green tea polyphenols into PCL/MWCNTs (multi-walled carbon nanotubes) has shown excellent anticancer activity against liver and lung cancers [24]. The encapsulation of oregano EOs into Poly(vinylidene fluoride) and PLCL/SF (Poly (l-lactic acid-*co*-ε-caprolactone)/Silk fibroin) revealed higher antioxidant and anticancer activities [25, 26]. Also, ginger oils loaded onto Poly(vinylidene fluoride) polymeric nanofibers showed superior antioxidant and anticancer properties [27]. Hence, EOs encapsulation into electrospun nanofibers could significantly improve their physicochemical properties and therapeutic activities.

*Pistacia lentiscus* var. *chia* belongs to the Anacardiaceae family, entailing at least eleven valued species. The fruits, resins, and other parts of different plant species of the Anacardiaceae family were extensively investigated. Further, their extracts have been widely utilized to target skin inflammations, gastrointestinal disorders, respiratory infections, wound healing, and several sorts of cancers. These promising therapeutic properties are owed to the rich content of these plants of various bioactive compounds such as triterpenes, monoterpenoids, oxygenated and non-oxygenated terpenes, polyphenols, etc. Therefore, all plant species of the Anacardiaceae family were rendered as promising antioxidants, anti-inflammatory agents, antimicrobials, and anti-cancers [58–66].

The EOs extracted from the resins of *P. lentiscus* (PLEO) showed unique anticancer properties. For instance, PLEO showed promising anticancer activity against colon cancers (Caco-2, HT29, and CT26 cells), suppressing cancer cells proliferation and decreasing Ki-67 protein expression and survival [67]. Also, PLEO suppressed Lewis lung carcinoma (LLC cells), inhibiting the expression of several inflammatory and angiogenic mediators (reduced VEGF and cytokines released by LLC cells, induced apoptosis, and inhibited angiogenesis) [68]. Further, PLEO suppressed Human leukemia cells (K562 cells), inducing antiproliferation and apoptosis and inhibiting angiogenesis via suppressing the release of VEGF from K562 cells [69]. Additionally, the EOs of *Pistacia atlantica* showed promising antitumor activity against breast cancer (SKBR3 and MDA-MB-231 cells), inducing cell cycle arrest and apoptosis [70, 71].

Despite these outstanding therapeutic properties, the ultimate benefits of natural extracts for clinical applications have been limited by their low solubility, reduced stability, and poor bioavailability. Specifically, the therapeutic efficacy of PLEO has been hindered by their poor stability, low solubility, poor bioavailability, and high volatility. Therefore, several nanocarriers have been exploited to maximize the biological activities of *P. lentiscus* extracts. Vrouvaki et al*.* [72] encapsulated PLEO into polymeric nanoparticles (PLA and PVA), showing excellent antimicrobial and anti-inflammatory properties against skin inflammation [72]. Furthermore, El-Chaghaby et al*.* [73] reported the biosynthesis of Ag nanoparticles using *P. lentiscus* leaves with promising antimicrobial activities against various strains of fungi and bacteria [73]. Moreover, Gortzi et al*.* [74] encapsulated the acidic and neutral fractions extracted from *P. lentiscus* gums into liposomes, depicting promising anticancer activity against liver (HepG2) and breast (MCF-7) cancer cells and antimicrobial activity against different strains of bacteria and fungi [74]. These findings support the remarkable biomedical properties of *P. lentiscus* extracts and encourage the future attempts to enhance their activities via their encapsulation inside other nanoformulations, particularly nanofibers attributing to the outstanding properties of nanofibers for EOs delivery.

In light of the above developments, this study aimed to extract the PLEO via a green extraction method of hydrodistillation and encapsulate the obtained PLEO into biodegradable and biocompatible PCL nanofibers (PCL-NFs). The PLEOs were selected based on their outstanding phytochemical composition and well-established biomedical properties. PCL-NFs were coloaded with 5FU alongside PLEO to assess their anticancer activities. The purpose of PLEO and 5FU encapsulation aimed to enhance their stability, release sustainability, targeting ability, and anticancer activity upon their local administration while reducing their side effects and off-target activity. Several characterization tests were performed on the prepared nanofibers to investigate their thermal stability, physical and mechanical integrity, biodegradability, and release behavior at physiological and acidic pH. Finally, the study aimed to investigate the antioxidant properties and anticancer activities of the obtained nanofibers against triple-negative breast cancer cells (MDA-MB-231), human adenocarcinoma breast cancer cells (MCF-7), and human skin melanoma cell line (A375).

## Materials and methodology

### Materials

#### Cell culture

Human triple-negative breast cancer cells (MDA-MB-231, ATCC HTB-26), Human adenocarcinoma cells (MCF-7, ATCC HTB-22), and human skin melanoma cell line (A375, ATCC CRL-1619) were acquired from ATCC (Wesel, Germany) and maintained in 75 cm^2^ flasks, containing Dulbecco's Modified Eagle Medium (DMEM) supplemented with Penicillin–Streptomycin (1%) and Fetal Bovine Serum (FBS) (10%). Consequently, cells were incubated in an atmosphere incubator (37 °C and 5% CO_2_).

#### Chemicals

PCL pellets (M.W. 80.000 g/mol) were purchased from Spectrum (Spectrum Chemical Mfg. Corp., Gardena, USA). Ethanol absolute was provided by CARLO ERBA Reagents group (Val de Reuil, France). Chloroform was purchased from Chem-Lab NV, Belgium. 5FU 99% was supplied by Alfa-Aesar, Thermo-Fisher (Kandel) GmbH, Germany. Phosphate Buffered Saline (PBS) (tablets) was provided by Genetix (Genetix Biotech, Asia, Pvt., Ltd.). KBr (FT-IR) was purchased from Merck (KGaA, Darmstadt, Germany). Natural Chios mastic gums (small tears), *P. lentiscus* variety *Chia*, were provided by Chios Gum Mastic Growers Association, Chios, Greece. 1, 1-diphenyl-2-picrylhydrazyl (DPPH) was supplied by Sigma (Sigma-Aldrich Co., Germany). Dimethyl sulfoxide (DMSO) was provided by Fisher Scientific (UK). Streptomycin-Penicillin mixture was provided by Lonza (Basel, Switzerland). Fetal bovine serum (FBS) and Dulbecco's Modified Eagle Medium (DMEM) were supplied by Gibco (Thermo-scientific, Regensburg, Germany). 3-(4,5-dimethylthiazol-2-yl)-2,5 diphenyltetrazolium bromide (MTT) was provided by Merck (KGaA, Darmstadt, Germany).

### Extraction of PLEO from the resins of *P. lentiscus*

PLEO was extracted from the resins of *P. lentiscus *via hydrodistillation, using a Clevenger apparatus, following the Mastic monograph accredited by the European Pharmacopeia (01/2008:1876) [75, 76]. The resins were first weighed (20 g), grounded into fine powder, added to 200 mL distilled water (inside a bottom-rounded flask), and then the flask was placed into a heating mantle. The heating mantle provided a persistent heat flow in order for the mixture in the flask to reach a constant boiling point between 95 °C and 105 °C. The steam carrying PLEO then condensed yielding two layers, separating the PLEO from the macerated water. Each 10 g of *P. lentiscus* powder needed 1 h of extraction, and thus each batch of extraction (20 g) was allowed to proceed for at least 2 h. Subsequently, the PLEOs were gathered and kept in opaque and tightly sealed amber glasses (at 4 °C). Multiple batches of extraction were ensued, achieving a final yield of 1% (w/w), where each 100 g of *P. lentiscus* powder yielded 1 g of PLEO.

### Preparation of PCL, 5FU-PCL, PLEO-PCL, and 5FU-PLEO-PCL Nanofibers

Four PCL polymeric solutions were initially prepared by dissolving PCL pellets 15% (w/v) in a solvent system of methanol and chloroform (MeOH:CHCl_3_; 50:50, v/v) [77, 78], and stirring (400 rpm) for 12 h in dark inside sealed containers, at room temperature. To prepare the PLEO-PCL polymeric solution, PLEO was slowly added to its corresponding PCL polymeric solution and then stirred (400 rpm) for 12 h in a sealed container, at room temperature, while being protected from light to eventually form a homogenous solution.

To prepare the 5FU-PCL polymeric solution, 5FU was added to its corresponding PCL polymeric solution and stirred (400 rpm) for 3 h at 60 °C, while being protected from light. Heating was provided to help dissolve 5FU in the solvent system and form a homogenous solution, and the obtained solution was consequently left to cool down at room temperature. The solution was then stirred (400 rpm) for 12 h in a sealed container, at room temperature while being protected from light.

Furthermore, to prepare the last polymeric solution of 5FU-PLEO-PCL, 5FU was first added to a PCL polymeric solution, stirred (400 rpm) for 3 h at 60 °C, and left to cool down at room temperature. Thereafter, PLEO was slowly added to the prepared solution forming a homogenous polymeric solution. Consequently, the prepared 5FU-PLEO-PCL solution was stirred (400 rpm) for 12 h in a sealed container, at room temperature while being protected from light.

Afterwards, the obtained solutions were electrospun using a vertical home built electrospinner supported by a high voltage power, at room temperature, in which the solutions could be introduced to a 5 mL plastic syringe installed into a syringe pump (SK-500II, Schenzhen Mindray Scientific Co., Ltd., China) and connected to a 21-gauge metallic needle via a Teflon tube. The developed nanofibers were collected on a stationary copper plate collector covered with an aluminum foil. The distance between the needle and the collector was 14.5 cm. Afterwards, to eliminate any solvent residues, the collected nanofibers were placed into a vacuum dryer for 48 h.

Several trials were initially conducted to prepare the nanofibers, before reaching the optimal parameters (Additional file 1: Table S1 and figures S1–S4). The optimal electrospinning conditions for the obtained nanofibers (PCL-NFs, PCL-NFs loaded with 0.4% 5FU, PCL-NFs loaded with 0.5% PLEO, and PCL-NFs co-loaded with 0.4% 5FU and 0.5% PLEO) were obtained at 4 mL/h flow rate and 20 kV.

The specified concentrations of both PLEO and 5FU used herein were chosen based on previous reports showing the effective anticancer activities of the resins and EOs extracted from different parts of *P. lentiscus* against different tumor tissues [41, 69, 79–82] and the effective antitumor activity of 5FU [12, 13, 52, 83].

### Characterization of nanofibers

#### GC–MS analysis of PLEO

The obtained components of the PLEO were chemically identified and analyzed using Agilent Technologies gas chromatography (GC–MS/7890B) coupled with a mass spectrometer detector (5977B), as reported previously [41, 84].

#### Morphological analysis and size determination of the nanofibers

The morphological surfaces of the nanofibers were examined utilizing a LEO Field Emission Scanning Electron Microscope (FE-SEM, Leo Supra 55). The nanofiber samples were fixed on aluminum stubs and their surfaces were further coated with a fine layer of Au (15 mA for 5 min of Au sputtering), under different magnifications. Au sputtering was performed to help enhance the nanofibers conductivity hence improve their observation. Several samples were selected randomly to determine the average diameter of the nanofibers, using ImageJ software.

#### Fourier transform Infrared spectroscopy (FT-IR) examination of the nanofibers

FT-IR spectroscopy (Nicolet 380 FT-IR, Thermo-Scientific, Madison, USA) was utilized in the spectral range of 4000–400 cm^−1^ (4 cm^−1^ resolution). The obtained FT-IR spectra aimed to examine the compounds structures, characteristic peaks, and verify the effective incorporation of the drugs loaded within the obtained nanofibers. For PLEO, a small drop of oil was spread on a piece of KBr window and placed directly in front of the IR beam. For 5FU and nanofibers, a tiny amount of pure 5FU in addition to small cuts of the nanofibers had initially been mixed with KBr (FT-IR grade). The obtained mixtures could then be compressed using a hydraulic press machine to form their corresponding small discs (15 T manual press machine, China). The small discs were then placed directly in front of the FT-IR beam [85–87].

#### Thermogravimetric analysis (TGA) of the nanofibers

A TGA Q50 thermogravimetric analysis device (TGA-Q50, TA instruments, New Castle, DE, USA) was used to investigate the thermal behavior and stability of the obtained nanofibers. Small samples of the nanofibers (~ 5 mg) were cut and projected to a heat range between 25 °C to 600 °C (heating rate 10 °C/min), under a nitrogen atmosphere with a flow rate of 50 mL/min. Consequently, the resulting weight loss for each sample could be recorded as a function of temperature. [88]

#### Nanofibers physical integrity study

Small areas of the prepared nanofibers (2 cm * 1.5 cm) were used to examine the physical integrity of the electrospun nanofibers. For this purpose, samples were soaked in distilled water in sealed containers (Falcon tubes sealed with parafilm strips) for 10 weeks at room temperature. Samples were then observed visually for their physical integrity and strength every 12 h for the first five time periods (12, 24, 48, 72, and 168 h) and then after 4, 8, and 10 weeks. The purpose of the visual inspection of the samples was to examine the intactness of the nanofiber samples and to make sure that no erosion or dissolution happened upon loading nanofibers with other compounds [89]. Also, for the topical drug delivery applications, it is crucial for the nanofibers to show good mechanical strength and physical integrity to ensure that the nanofibers mats could demonstrate consistent adherence and withstand high mechanical pressure upon application to biological tissues, and provide sustainable and local delivery of their cargos [89–92]. Therefore, both physical integrity and tensile strength studies have been conducted. All samples were tested in triplicate.

#### Nanofibers tensile strength test

Nanofibers tensile characteristics were investigated utilizing a tensile TS1500 testing machine (TS1500 1500N, TSL solutions, Acutance Scientific, Tunbridge Wells, Kent, UK). Random samples were chosen in triplicates (2 mm × 10 mm) and tested employing a cross-head section speed of 0.35 mm/min (Initial length: 10 mm; Load force: 5 N) [93].

#### Nanofibers XRD investigation

To examine the X-ray diffraction patterns and the crystalline structures of the obtained pristine and loaded nanofibers, an X-ray diffractometer (D8 advance X-ray diffractometer model 4040, Bruker, USA) was utilized with Cu Kαλ = 0.1540600 radiations over a 2θ range of 5–80° (49 kV/ 30 mA) [94]. The XRD patterns provided more in-depth comprehension on the effect of loading and co-loading bioactive compounds in the polymeric structure of the PCL nanofibers.

### Nanofibers biodegradability study

The *in-vitro* biodegradability behavior of the obtained nanofibers was investigated by studying the weight loss change of the nanofibers in PBS solution (pH 7.4) at 37 °C inside an incubating shaker (120 rpm). For this purpose, several samples of the obtained nanofibers were cut into 2.25 cm^2^ (1.5 cm * 1.5 cm) and 3.0625 cm^2^ (1.75 cm * 1.75 cm) surface areas, with an average weight ranged between 10 and 16 mg. All samples were carried out in triplicate. Consequently, samples were submerged in PBS solution (pH 7.4) and were then placed in an incubating shaker (120 rpm and 37 °C) for different time periods up to 42 days. Time periods were predetermined at 0, 3, 7, 14, 28, 35, and 42 days. At the end of each time interval, samples were removed from the PBS solution and washed with distilled water to remove any minerals that could be deposited by the PBS solution [95, 96]. For the weight loss study, samples were dried at 50 °C, and the weight loss percentage of the samples was calculated using the following equation:

1 $$Weight\;loss\left( \% \right) = \frac{{Wi - Wf}}{{Wi}} \times 100$$

Wi: initial weight recorded of the nanofiber sample (mg).

Wf: final weight recorded of the nanofiber sample (mg).

### Nanofibers release study

Nanofibers *in-vitro* release profiles of 5FU and PLEO were investigated for 7 days (168 h) in release media contain PBS solutions at pH values of 7.4 and 5.4, separately. Briefly, 5 mg cuts of the nanofibers were soaked in 10 mL of the prepared release media (pH 7.4 and 5.4) and placed in an incubating shaker (120 rpm and 37 °C). All samples were carried out in triplicate. Subsequently, the release kinetics of 5FU and PLEO could be examined, utilizing a UV–Vis double beam spectrophotometer, at 266 nm and 256 nm, respectively, (Cary 3500 UV–Vis Engine, Agilent Technologies Australia (M) Pty Ltd, Mulgrave, Australia). The UV–Vis measurements were based on calibration curves performed on both 5FU and PLEO. The calibration curves for 5FU were established in PBS solutions at pH 7.4 (y = 0.0595x + 0.0123, R2 = 0.9999) and at pH 5.4 (y = 0.0607x—0.0054, R2 = 0.9997). Also, the calibration curves for PLEO were prepared in PBS solutions at pH 7.4 (y = 0.004x + 0.0607, R2 = 0.9912) and at pH 5.4 (y = 0.0026x + 0.0257, R2 = 0.9966). At predetermined time intervals, 3 mL of the release media were withdrawn, and their corresponding absorbance values were spectrophotometrically read. Subsequently, the released amounts of both compounds were calculated using Beer Lambert Law (Eq. 2). The cumulative release percentages were determined (using Eq. 3) and plotted [93, 97].

2 $$A=\varepsilon bc$$

A: absorbance.

$$\varepsilon :$$ absorptivity coefficient.

b: path length of radiation passing through the cuvette.

c: sample concentration.

3 $$Cumulative\; release \left(\%\right)= \frac{Amount\; released}{Initial\; amount}\times 100$$

Additionally, different mathematical kinetic release models were developed to further investigate the release mechanisms of 5FU and PLEO. [98–100]

### Nanofibers antioxidant activity

To evaluate the antioxidant activity of the electrospun nanofibers, an antioxidant assay using 1, 1-diphenyl-2-picrylhydrazyl (DPPH) was performed. Small cuts of the nanofibers (5 mg per each) were immersed in 3 mL methanolic solution of DPPH (0.12 mM). Samples were then incubated in darkness for 30 min, at room temperature. Then, the absorbance of the obtained mixtures could be measured at 517 nm, using FLUOstar microplate reader (BMG Labtech, Germany), where 200 $\mu$ L aliquots of the mixtures were placed in a 96 well-plate, in triplicate. The percentage of DPPH scavenging was calculated using the following Eq. 4, where Ab and As refer to the absorbances of the blank (DPPH methanolic solution) and the sample mixture measured at 517 nm, respectively: [97]

4 $$\mathrm{\%}DPPH scavenging=\left[\frac{{A}_{b}-{A}_{s}}{{A}_{b}}\right]\times 100$$

### Nanofibers anticancer activity

#### MTT-assay

The anticancer activity of 5FU, PLEO, and nanofiber groups (PCL, 5FU-PCL, PLEO-PCL, and 5FU-PLEO-PCL nanofibers) was investigated in vitro against human breast cancer cell lines, MDA-MB-231 and MCF-7, and against human melanoma cancer cell line, A375 cells, utilizing the MTT-assay (3-[4,5-dimethylthiazol-2-yl]-2,5-diphenyl tetrazolium bromide), as described previously with some modifications [93, 97]. For this purpose, the cancer cells of each cell line had been initially seeded in a 96 well-microplate at a density of 5 × 10^3^ viable cells per well and incubated for 24 h (37 °C and 5% CO2). Subsequently, the nanofibers were UV-sterilized for 30 min, weighed, and cut to give a range of concentrations (0.01, 0.1, 1, 10, 100, and 1000 μg/mL) of 5FU and/or PLEO which were added to the 96-well plates with each well containing cultured cells and 100 μL of medium. Cells were further incubated for 72 h. Thereafter, 20 μL aliquots of the prepared MTT solution (500 μg/mL) were added to the wells, and the plates were then incubated for 4 h, at 37 °C. Afterwards, 150 μL of DMSO was added to each well after removing the medium to help dissolve formazan crystals in the last part. Following that, a FLUOstar microplate reader (BMG Labtech, Germany) was used to read the absorbance at 570 nm and 630 nm wavelengths, and the cell viability could be reported utilizing the following equation:

5 $$\% {\mathrm{cell\;viability}} = \left[ {\frac{{\text{OD}_{{\text{experimental}}}^{{570}} }}{{\text{OD}_{{\text{control}}}^{{570}} }}} \right] \times 100$$

Moreover, to investigate any possible interference of the PLEO, 5FU, or their combination with the MTT compound, MTT was added to these compounds and compared to an MTT positive control (MTT added to cultured HSF normal cells), as previously reported with some modifications [101]. For the test, an MTT solution (0.5 mg/mL), 5FU (4 mg/mL), and PLEO (5 mg/mL) were prepared in PBS. Consequently, using a 96 well-microplate, several aliquots of 10 μL of the prepared MTT solution were added to 100 μL of 5FU, 100 μL of PLEO, and 100 μL of 5FU and PLEO (50:50). The microplate was then incubated for 4 h in dark at 37 °C. Afterwards, 100 μL of DMSO were added to the wells and the plate was further incubated for 10 min and shaken before reading absorbance at 570 nm using FLUOstar microplate reader (BMG Labtech, Germany). All samples were performed in triplicate.

### Statistical analysis

Results and data were reported as mean ± standard deviation (SD). Also, all formulations were prepared in three replicates. One-way analysis of variances (ANOVA analysis) was utilized to determine statistical differences using GraphPad Prism 8 software, and a P value of ≤ 0.05 was selected to indicate statistically significant differences.

## Results and discussions

### Analysis of PLEO using GC–MS

The GC–MS analysis conducted on the collected PLEO identified 29 chemical compounds [41]. The primary compounds were α-pinene (81.20%), β-Myrcene (4.70%), β-Pinene (2.97%), and 3-Carene (1.11%), which belong to the monoterpenes group. The GC–MS study also identified several compounds entailed under the sesquiterpenes, oxygenated sesquiterpenes, phenylpropanoids, and oxygenated monoterpenes chemical groups. These results were consistent with previously published reports which showed similar findings related to the major compounds extracted from the resins of *P. lentiscus* of which α-pinene was the major component of PLEO [75, 102], verifying the composition of PLEO.

Previous reports showed that thirty-seven compounds belonging to the monoterpenes group have promising anticancer properties [103]. α- Pinene, the prime monoterpene reported in PLEO by GC–MS, exhibited remarkable anticancer activities against various tumors, including prostate cancer and hepatocellular carcinoma [104–106]. Also, α-pinene was reported to induce apoptosis and cell-cycle arrest (G2/M) in hepatocellular carcinoma and ovarian cancer [107, 108]. Additionally, α-pinene could suppress cancer invasion by inhibiting matrix metalloproteinase-9 expression in breast cancer [109] and activate natural killer cells (lymphocytes), increasing their cytotoxicity against colon cancer (CT-26) [110].

### Morphological analysis and size determination

**Fig. 1:**
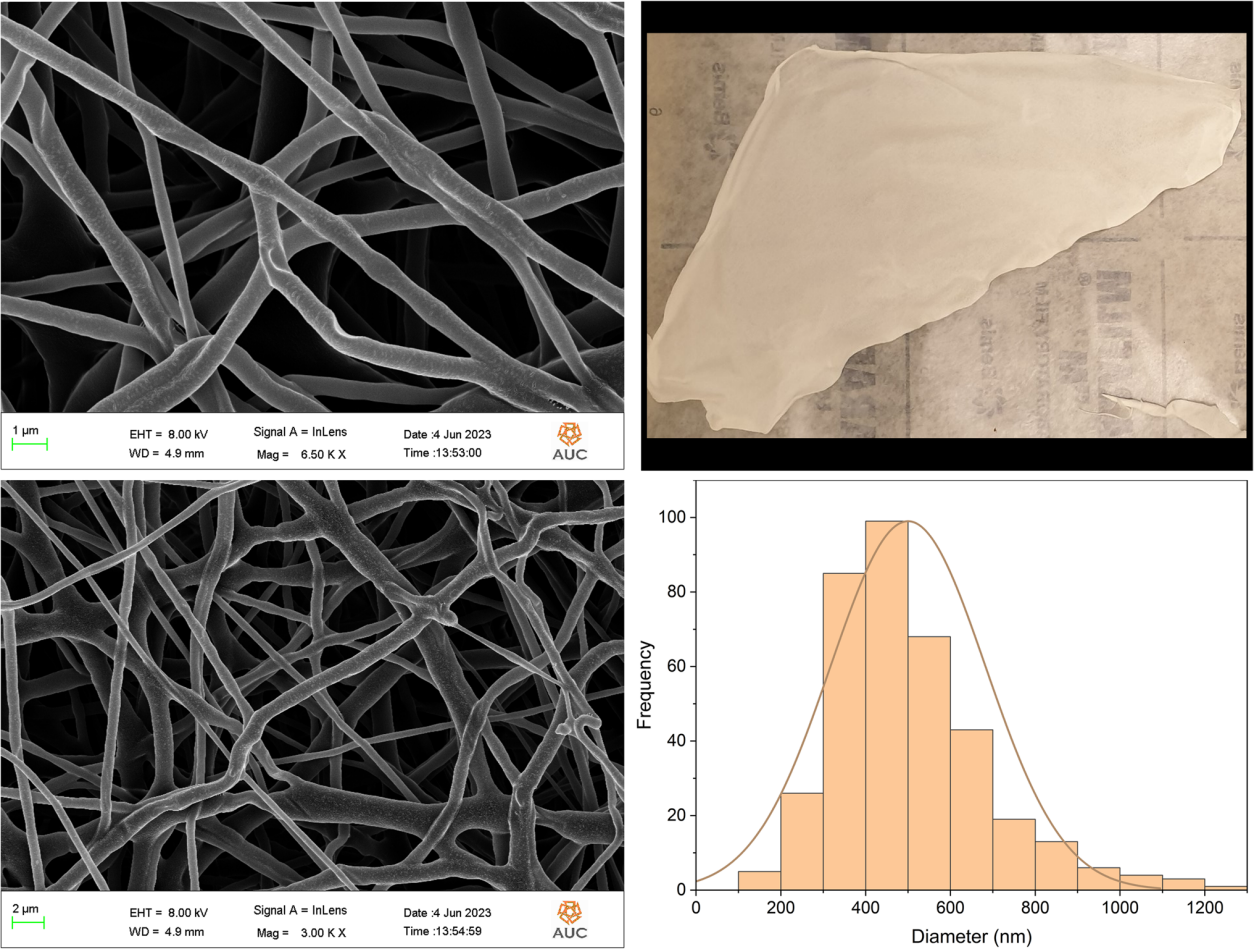
PCL-NFs

**Fig. 2:**
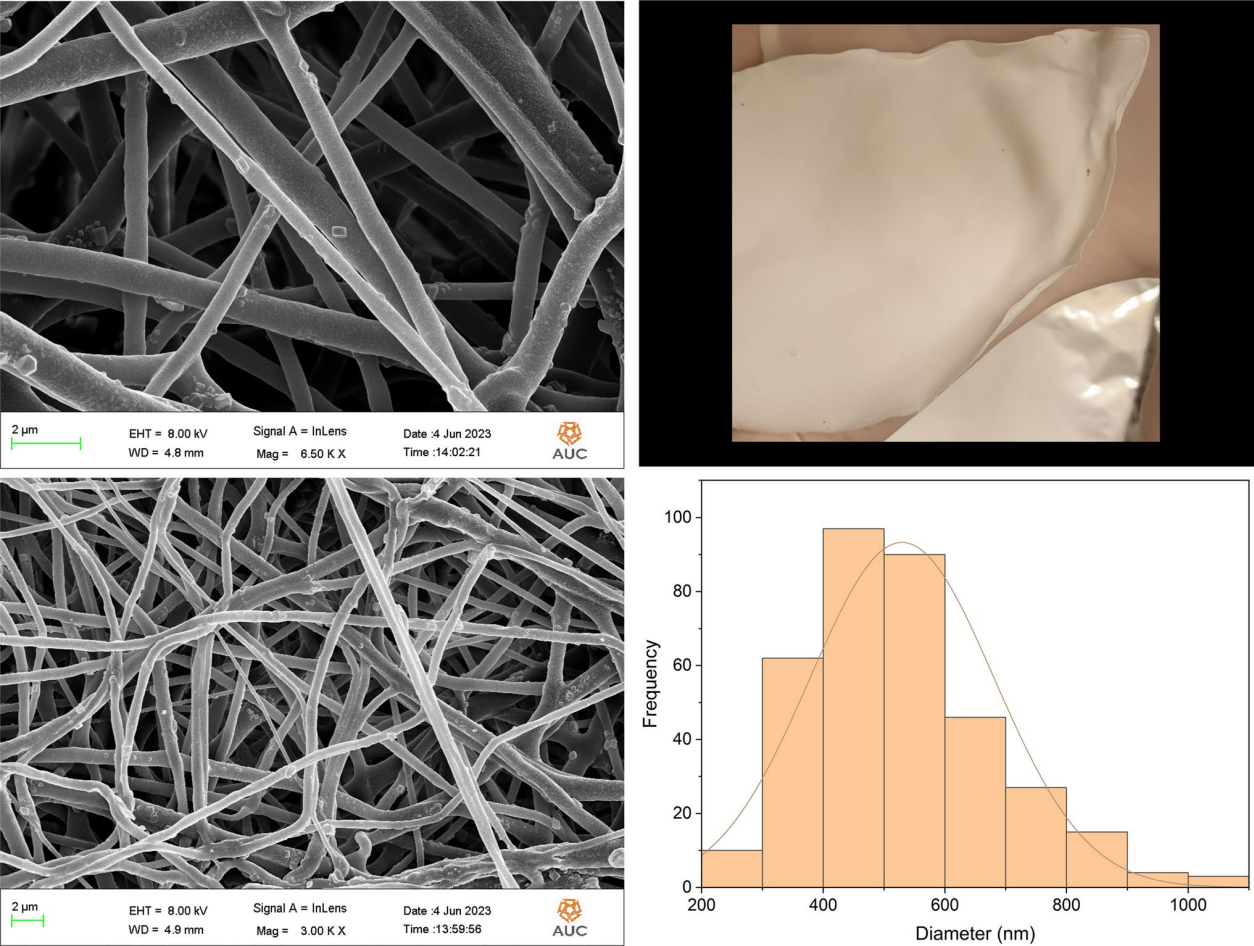
5FU-PCL-NFs

**Fig. 3:**
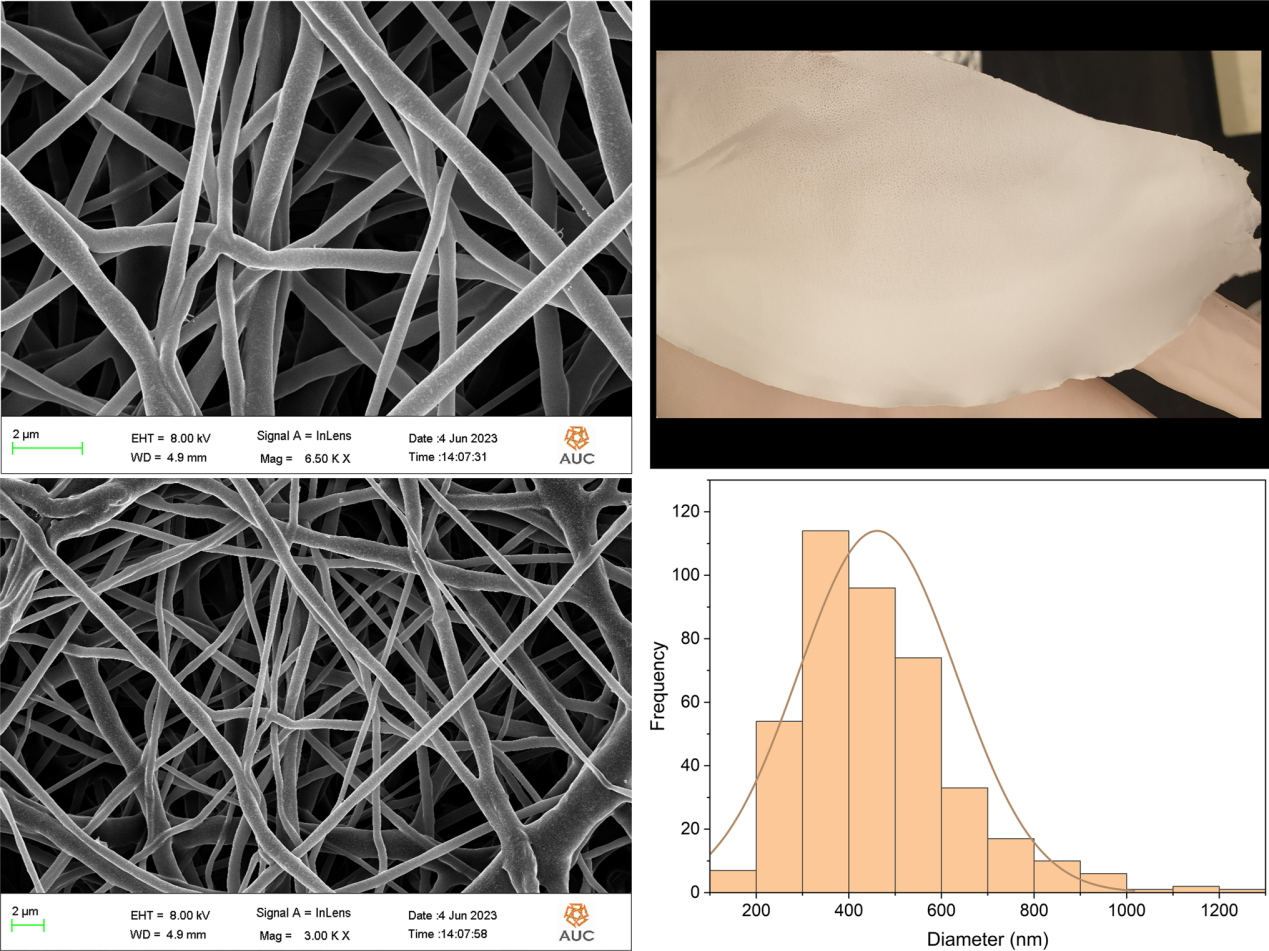
PLEO-PCL-NFs

**Fig. 4:**
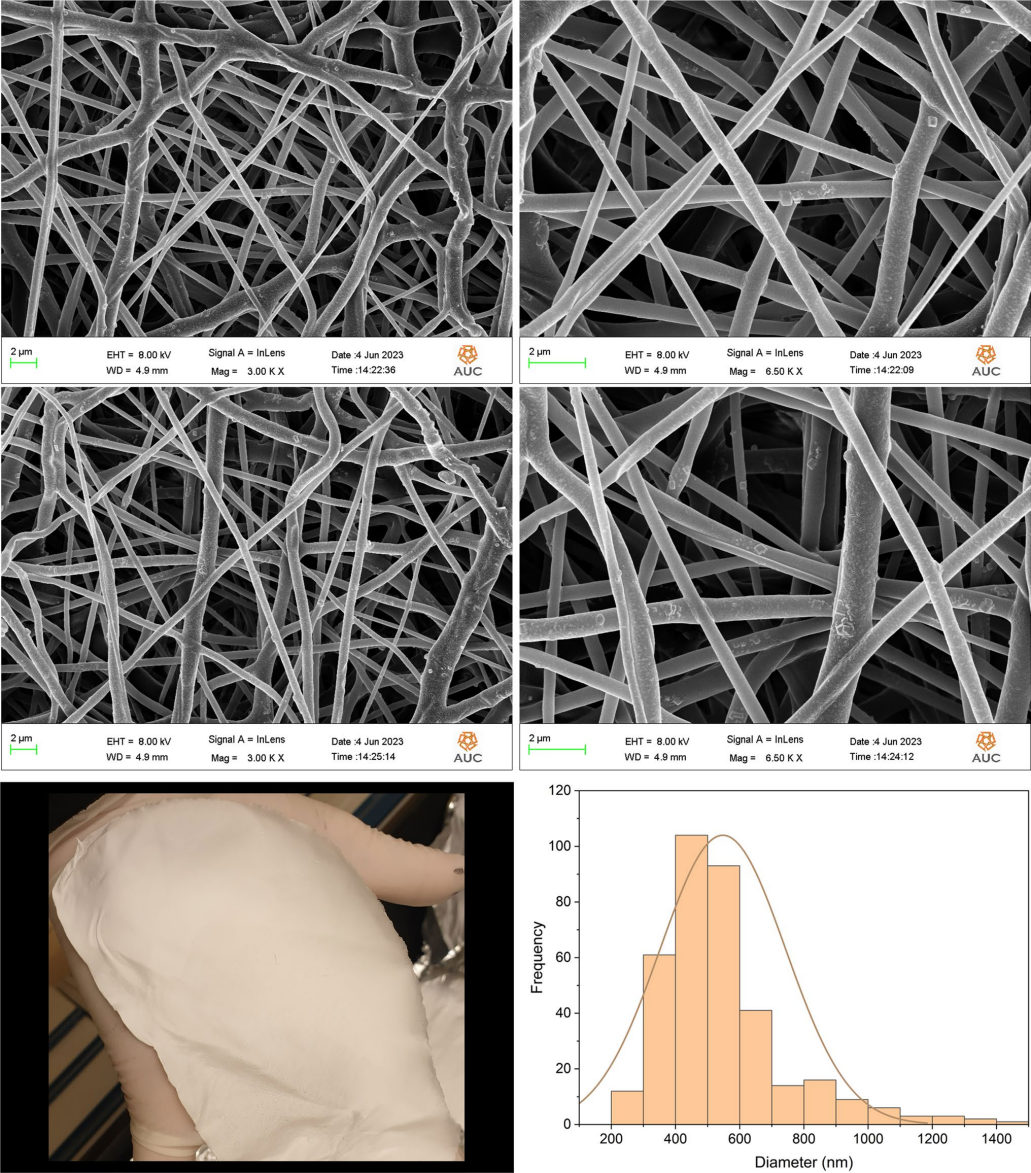
5FU-PLEO-PCL-NFs

The morphological appearance of the PCL, 5FU-PCL, PLEO-PCL, and 5FU-PLEO-PCL nanofibers was investigated utilizing FE-SEM. Notably, several trials were conducted to prepare the nanofibers, before reaching the above parameters, as can be seen in Additional file 1 (supplementary Table S1 and Figs. S1–S4). The fiber mats could be observed with no adhesion or beads, as can be seen in Figs. 1, 2, 3, and 4. Additionally, the size distribution/average diameter of the obtained nanofibers was in the range of 290.71 nm to 680.95 nm. (PCL-NFs: 499.75 nm ± 182.86 nm; 5FU-PCL-NFs: 529.56 nm ± 151.39 nm; PLEO-PCL-NFs: 461.24 nm ± 170.53 nm; 5FU-PLEO-PCL-NFs: 547.41 nm ± 195.59 nm).

The obtained nanofibers had diameters below one μm, resulting in larger surface areas and more significant interactions with treated tissues [111], allowing for better tumor cell penetration of the released compounds.

### FT-IR analysis of the nanofibers

**Fig. 5:**
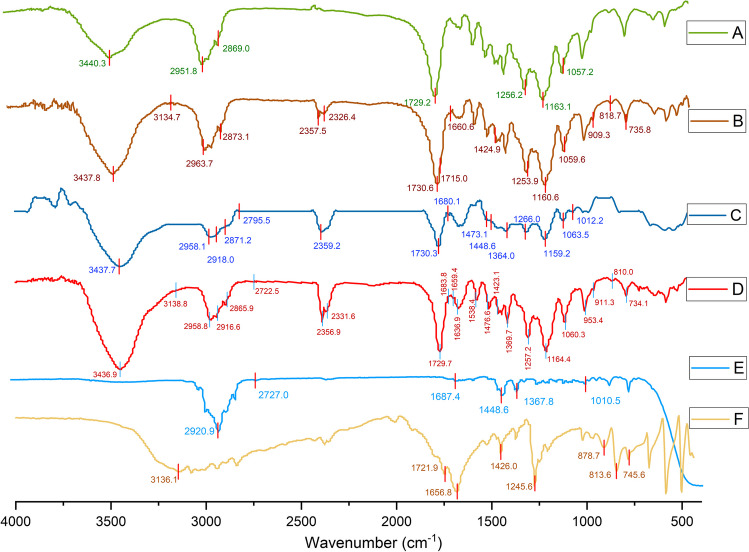
FTIR spectra of PCL-NFs **A**, 5FU-PCL-NFs **B**, PLEO-PCL-NFs **C**, 5FU-PLEO-PCL-NFs **D**, PLEO **E**, and 5FU **F**

The chemical structures of the PCL, 5FU-PCL, PLEO-PCL, and 5FU-PLEO-PCL nanofibers were examined utilizing the FT-IR spectrum of each nanofiber. In addition, the FT-IR spectra of 5FU and free PLEO were also investigated (Fig. 5). For PCL-NFs, the FT-IR spectrum (Fig. 5A) exhibited a sharp peak at 1729.2 cm^−1^ region and another characteristic peak at 3440.3 cm^−1^ region, corresponding to the stretching vibration of the ester carbonyl group (C=O) and hydroxyl group (–OH), respectively. [112, 113] Other characteristic peaks of PCL could be seen at 2951.8 cm^−1^ and 2869.0 cm^−1^, corresponding to the asymmetric and symmetric stretching vibrations of (-CH2) groups, respectively. [112, 113] Additionally, the stretching vibration of the ester groups (–C–O–) could be shown with peaks revealed at 1057.2 cm^−1^, 1163.1 cm^−1^ (symmetric stretching), and 1256.2 cm^−1^ (asymmetric stretching) [113].

For 5FU, several peaks were depicted (Fig. 5F), confirming the chemical structure of 5FU, including two peaks at the 3136.1 cm^−1^ and 1721.9 cm^−1^ regions which are related to the stretching vibrations of the bonds presented in (N–H) and (C=O) groups, respectively. Additionally, a peak was shown at the 1656.8 cm^−1^ region referring to the (C=C) and (C=O) groups. Other peaks shown at 1245.6 cm^−1^ and 1426.0 cm^−1^ regions refer to the stretching vibrations of the (C–N) and (C–F) groups, respectively. The out of plane bending vibration of the (C-H) group was detected at 878.7 cm^−1^, whereas the deformation vibration of the out of the plane (C–H) group could be revealed at 813.6 cm^−1^ and 745.6 cm^−1^. [114, 115] For 5FU-PCL-NFs (Fig. 5B), the corresponding FT-IR spectrum showed the peaks related to 5FU (3134.7 cm^−1^, 1660.6 cm^−1^, 1715.0 cm^−1^, 1424.9 cm^−1^, and 1253.9 cm^−1^) and PCL (3437.8 cm^−1^, 2963.7 cm^−1^, 2873.1 cm^−1^, 1730.6 cm^−1^, and 1160.6 cm^−1^) with some shifting noticed for certain peaks. The increased intensity and the shifts noticed might be explained by the superimposition exerted by the PCL NFs peaks on the 5FU peaks [90]. However, as evident from the FT-IR spectra of 5FU and PCL NFs, no significant alterations in the chemical structure of 5FU were observed upon loading in the PCL NFs [113–115]. Similarly, Fig. 5D depicted the presence of the peaks related to both 5FU and PCL in the developed 5FU-PLEO-PCL-NFs.

For PLEO (Fig. 5E), the FT-IR spectrum showed two peaks at 2920.9 cm^−1^ and 2727.0 cm^−1^ regions, referring to the bond stretching of the (CH) group. Another peak was revealed at 1687.4 cm^−1^ which refers to the (C=O) stretching in the aldehyde group. Also, two peaks could be shown at 1448.6 cm^−1^ and 1367.8 cm^−1^, indicating the deformation modes of the (CH2) groups, and another peak was recorded at 1010.5 cm^−1^ which refers to the bond stretching in (C-O) group [116]. For PLEO-PCL-NFs (Fig. 5C), the corresponding FT-IR spectrum revealed all the peaks related to PLEO (2918.0 cm^−1^, 1680.1 cm^−1^, 1448.6 cm^−1^, 1364.0 cm^−1^, 1012.2 cm^−1^) and PCL (3437.3 cm^−1^, 2958.1 cm^−1^, 2871.2 cm^−1^, 1730.3 cm^−1^, 1159.2 cm^−1^) with some shifting noticed for certain peaks. Also, the higher intensity and broadening observed for certain peaks could be interpreted by the superimposition effect exerted by the PCL peaks [90]. Alternatively, the complexity of the PLEO components which contain multiple hydroxyl groups might affect the intensity of the resulting peaks and, more importantly, might have developed inter-/intramolecular hydrogen bonds [90]. However, the overlapping of the FT-IR spectra for both indicates that there are no significant interactions or alterations in the chemical structure of the obtained PLEO-PCL-NFs [113, 116]. Similarly, Fig. 5D depicted the presence of the peaks related to both PLEO and PCL in the developed 5FU-PLEO-PCL-NFs.

### Thermogravimetric analysis (TGA) examination of the nanofibers

**Fig. 6:**
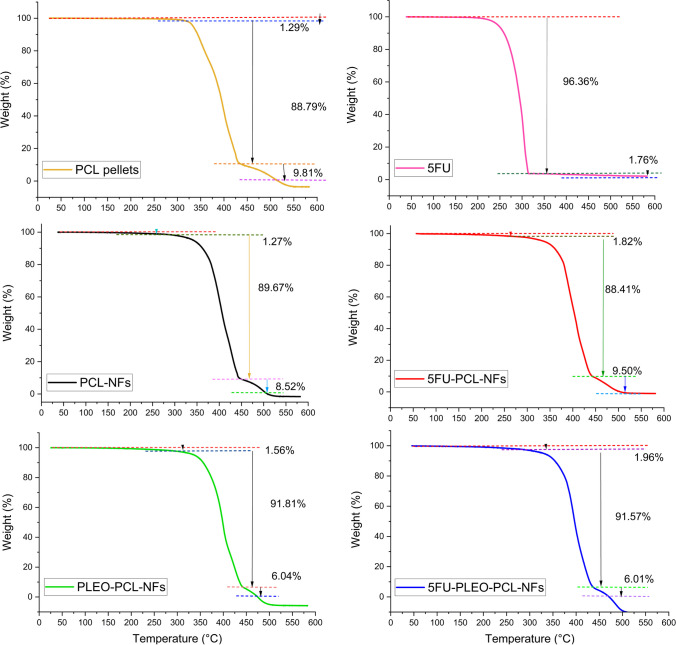
TGA curves of PCL pellets, 5FU, PCL-NFs, 5FU-PCL-NFs, PLEO-PCL-NFs, and 5FU-PLEO-PCL-NFs

**Fig. 7:**
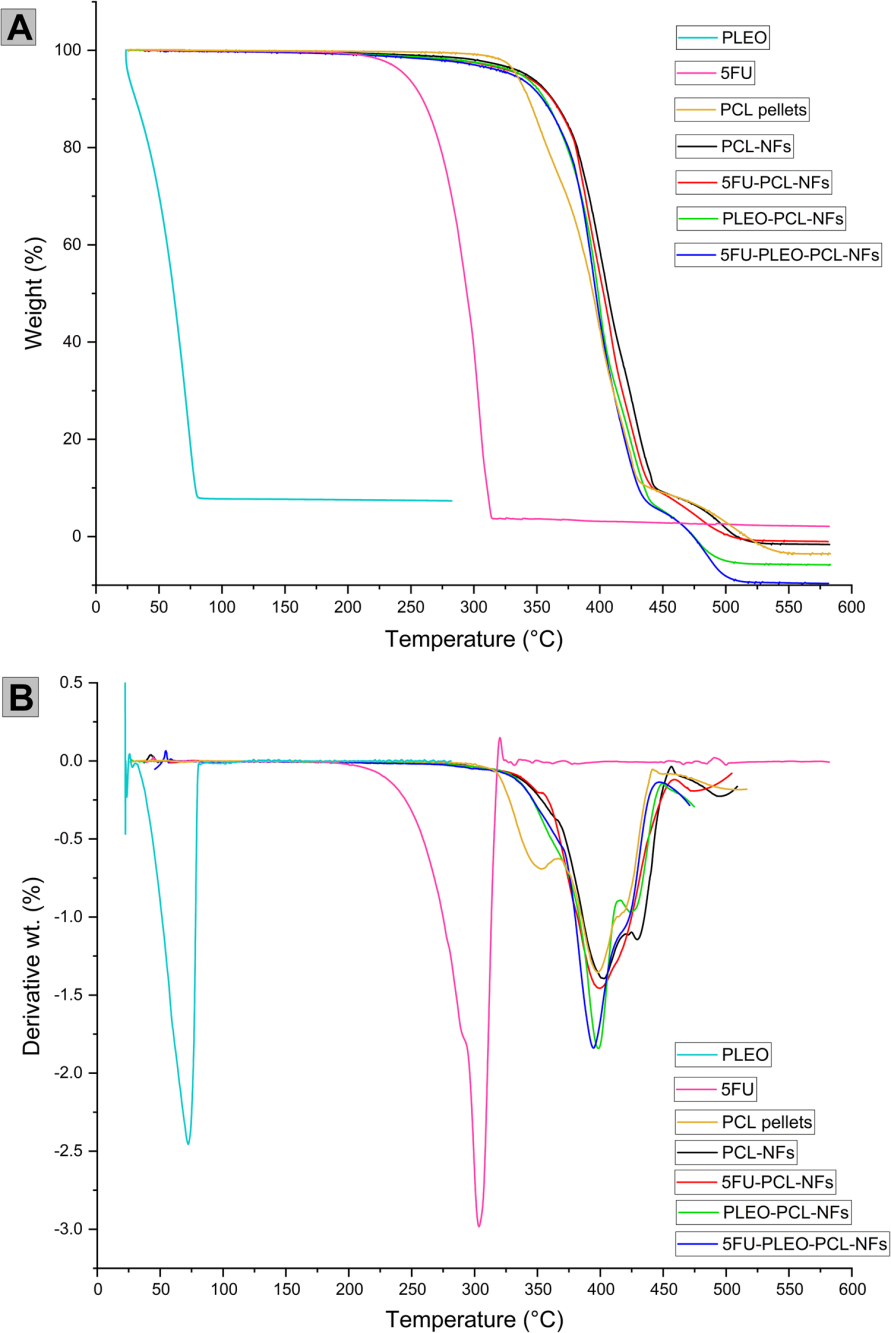
TGA **A** and DTG **B** curves of PCL pellets, PLEO, 5FU, PCL-NFs, 5FU-PCL-NFs, PLEO-PCL-NFs, and 5FU-PLEO-PCL-NFs

The TGA analysis of the samples was performed up to 600 °C as can be seen in Figs. 6 and 7. The thermogram of PCL pellets revealed two major stages of weight loss. The first decay was initiated at *ca.* 325 °C, with 18.55% loss of the sample weight, whereas the second decay was shown at *ca.* 356.9 °C, with 70.16% weight loss, and the last point of decomposition could be recorded at 516.46 °C. This pattern of decomposition of the polycaprolactone pellets refers to its good thermal stability which can be attributed to the aliphatic polyester groups in the backbone of PCL [117, 118]. In addition, the electrospun nanofibers of PCL showed a good thermal stability as well with one major stage of weight loss of 89.67% which started at 301.6 °C, and the final decomposition could be detected at 508.88 °C. Similarly, the aliphatic polyester groups in the PCL backbone can explain such thermal behavior in addition to the semi-crystalline structure of the obtained PCL nanofibers which might further be associated with the greater uniformity of the presented thermal behavior [117, 119].

For free 5FU, the corresponding thermogram showed one rapid stage of decomposition (96.36%) within a narrow temperature range (200 to 303.64 °C) which agrees with previous reports [120]. However, the thermogram of the PCL nanofibers loaded with 5FU showed better thermal stability, with a prolonged decomposition rate that started at 318.05 °C and ended at 448.83 °C for the major first weight-loss stage (88.41%). A second stage of 9.50% weight loss was then observed ending at the last point of decomposition at 504.59 °C. The higher and better thermal stability revealed with such behavior can certainly be assigned to the rigid structure of the electrospun PCL nanofibers blending 5FU in their backbone [117, 119, 121].

For free PLEO, the corresponding thermogram showed a major weight loss of 91.84% at 84.7 °C. In contrast, the thermograms of PLEO-NFs and 5FU-PLEO-NFs revealed almost similar behaviors of the thermal stability that was shown with the 5FU loaded nanofibers, with a prolonged decomposition starting within the temperate range of 290 °C to 310 °C for the first major stage of weight loss (91.81% and 91.57%), followed by a second weight-loss stage (6.04% and 6.01%), and ending with the final decomposition temperature of 474.64 °C and 471.07 °C for the PLEO-PCL-NFs and 5FU-PLEO-PCL-NFs, respectively. Although the electrospun nanofibers blended with the above-mentioned compounds could reveal higher thermal stability compared to the free compounds, the thermal stability of the blended nanofibers was slightly decreased compared to the unloaded nanofibers (PCL-NFs) as can be shown in Figs. 6 and 7 and as was illustrated earlier with the lower decomposition points of the blended nanofibers compared to non-blended ones. This decrease might be negligible and could be explained by the low amounts of the compounds blended and their lower thermal stabilities.

The remarkable thermal stability of the loaded nanofiber mats is crucial for their local application and attachment to biological tissues and imparts higher stability for the loaded compounds, supporting their sustained release, especially in tumor environment which is typically characterized by hyperthermia [39, 52, 119]. PLEO is known to have poor thermal stability, similar to other EOs, in which several bioactive components might break down [41]. Hence, the observed thermal stability supports the physicochemical and therapeutic efficacies of the blended bioactive compounds in a biocompatible and biodegradable nanosystem, ensuring their local delivery to the targeted tumor tissues.

### Nanofibers physical integrity study

To determine the intactness and the rigidity of the obtained nanofibers, the physical integrity study was performed as well as to provide an indication for any dissolution or disruption signs which could be revealed by the visual observation of the samples immersed in distilled water for 10 weeks. [89] All samples showed good mechanical and physical integrity, where no disintegration or dissolution could be noticed, and the samples showed compact and intact shapes without any signs of deformation or disruption in their structures. Such properties provide superior benefits for the local delivery applications, reinforcing the adherence of the developed nanofibers to biological tissues and their surfaces, augmenting the local delivery of their loaded compounds and drugs [89, 91, 92]. Furthermore, it is worth noting that the excellent integrity shown in addition to the stable and rigid structures of the obtained nanofibers can be attributed to the semi-crystalline structure of the PCL polymer which could further be enhanced upon blending of the PCL with 5FU and/or PLEO, in which several hydrogen bonds (intra- and inter-molecular hydrogen bonds) between these compounds could be developed enhancing the overall physical integrity of the nanofibers [122–124].

Hence, the observed physical integrity of the nanofibers guarantees their ability to withstand high mechanical pressures and stably attach to their targeted tumor tissues thus securing sustained delivery of their loaded compounds [89–92].

### Nanofibers tensile strength test

Nanofibers mechanical properties are depicted in Table 1. Interestingly, the addition of PLEO and/or 5FU significantly changed the nanofibers mechanical characteristics. The tensile strength of PLEO-PCL-NFs was decreased. A reasonable interpretation for this decrease might be attributed to the induced reduction in the physical interactions among the polymeric chains of the PLEO-PCL-NFs. Conversely, the tensile strength of the 5FU-PCL-NFs was improved which might be explained by the reinforced integrity obtained upon the blending of the 5FU crystalline molecules into the PCL backbone structure. More importantly, the nanofibers loaded with both 5FU and PLEO showed the highest increase in the tensile strength properties, which could be ascribed to the greater compatibility obtained between the 5FU and PLEO components showing enhanced integration and incorporation with the polymeric chains and structural background of the PCL-NFs. These findings are supported by previous studies [125].

**Table 1:** Mechanical properties of the nanofibers

	Yield strength (MPa)	Ultimate tensile strength (MPa)	Young’s modulus (MPa)	Elongation at breaking point
PCL-NFs	21.63 ± 1.56	49 ± 0.96	111.74 ± 3.58	1.25 ± 0.11
5FU-PCL-NFs	27.84 ± 0.99	62.43 ± 2.71	207.59 ± 14.01	1.63 ± 0.003
PLEO-PCL-NFs	17.84 ± 1.02	40.78 ± 1.96	74.19 ± 18.24	ND*
5FU-PLEO-PCL-NFs	33.65 ± 2.89	70.57 ± 0.84	300.72 ± 32.46	1.58 ± 0.02

^*^Not determined as PLEO-PCL-NFs did not reach a breaking point under the test conditions

Interestingly, the observed high physical integrity and tensile strength of the obtained nanofibers support their consistent prolonged attachment during the healing processes and ability to deliver the loaded compounds [89–92, 125].

### Nanofibers XRD investigation

**Fig. 8:**
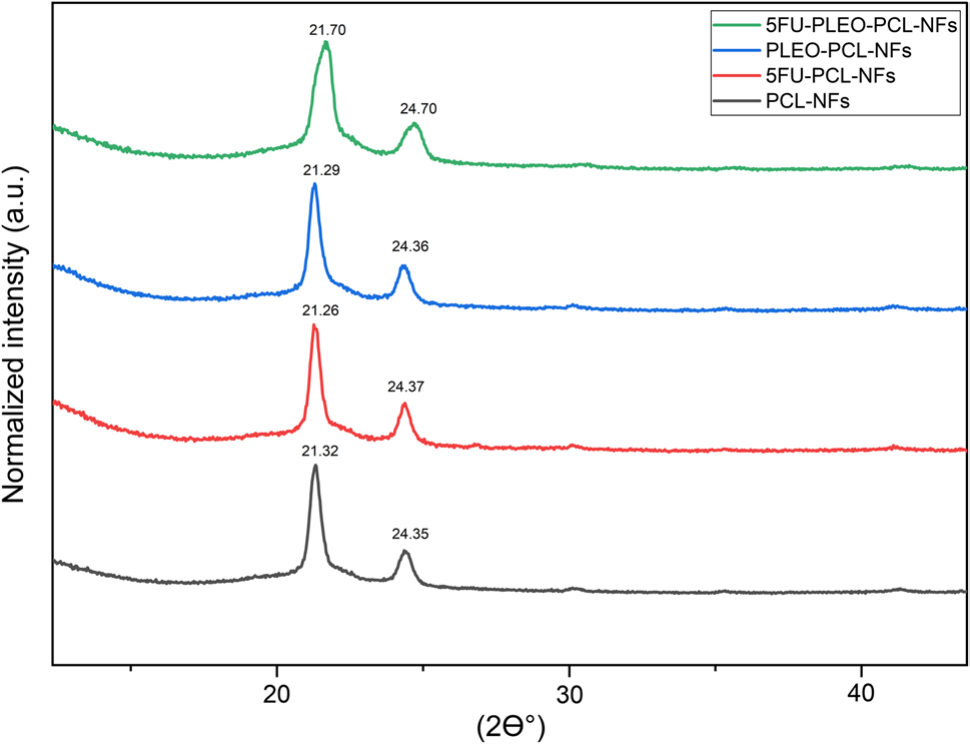
XRD patterns of PCL-NFs, 5FU-PCL-NFs, PLEO-PCL-NFs, and 5FU-PLEO-PCL-NFs

The crystalline structure of the prepared nanofibers was examined via the X-ray diffraction analysis of the nanofiber groups over a 2θ range of 5–80°, and the resulting patterns are shown in Fig. 8. The two characteristic diffraction peaks that can be observed among all the nanofiber groups are attributed to the semicrystalline nature of the PCL polymer which is evident at 2θ values of 21.32° and 24.35°, corresponding to the lattice planes (110) and (200), respectively. [126] Despite the minor shifts shown by the two peaks, their consistent appearance in all other nanofiber groups confirms the maintained crystallinity of the PCL polymeric structure upon loading 5FU, PLEO, and the combination of both. More importantly, the XRD patterns of 5FU-PCL-NFs and 5FU-PLEO-PCL-NFs revealed no characteristic diffraction peaks related to the 5FU crystalline structure. This might be attributed to the incorporation and entrapment of 5FU inside the PCL polymeric nanofibers with negligible traces remained on the external surface of the developed fibers [127]. Another reason might explain these findings is the insufficient time provided, during the rapid process of electrospinning, for the polymer and other compounds to develop well-organized crystalline structure [94]. However, the decrease in the sharpness of the PCL characteristic peaks in the XRD patterns of the loaded nanofibers might refer to the relatively disturbing effect of the loaded compounds on the crystalline structure of PCL. The two peaks in the XRD pattern of 5FU-PLEO-PCL-NFs shifted from 21.32° and 24.35° to 21.70° and 24.70°, respectively, which could be influenced by the 5FU crystalline structure.

Hence, the crystalline structure of the PCL nanofibers was maintained following their loading with 5FU and PLEO. This signifies the stability of the nanofibers and consequently their ability to deliver the loaded compounds [117, 127].

### Nanofibers biodegradability study

**Fig. 9:**
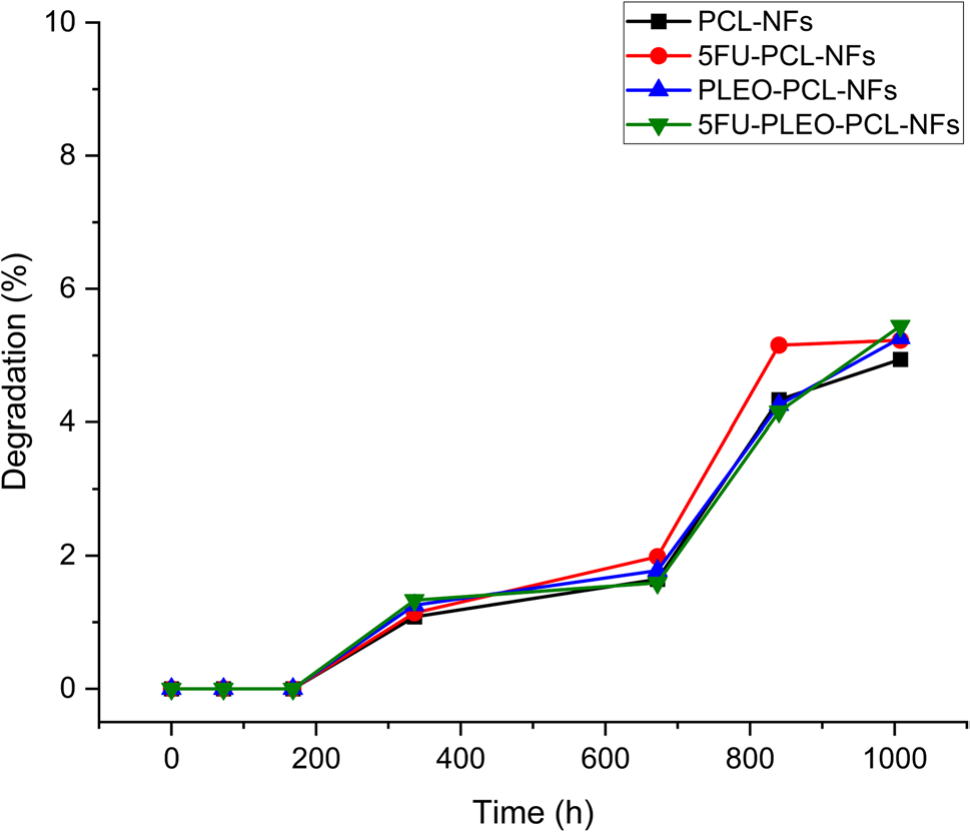
Degradation behaviors of PCL-NFs, 5FU-PCL-NFs, PLEO-PCL-NFs, and 5FU-PLEO-PCL-NFs

The biodegradability of the electrospun nanofibers was investigated over 42 days in PBS solution, where the weight loss and degradation behavior of the nanofibers were examined as illustrated in Fig. 9. All nanofibers showed comparable rates of weight loss. This can be explained by the presence of the hydrophobic and semi-crystalline PCL polymer as the main backbone of the electrospun mats with the poly and long aliphatic chain groups in the PCL structure, providing slow and prolonged degradation rates for the obtained nanofibers, as reported previously [119]. Moreover, the degradation study gave an indication of the hydrolytic process which might have occurred to the PCL polymeric molecules, in which the ester bonds present in the PCL chains underwent a slow hydrolysis, resulting in a rise of the hydroxyl and carboxyl groups in the backbone structure of the nanofibers, leaving the nanofibers vulnerable to a slow and a steady process of degradation. Furthermore, compared to free PCL nanofibers, a slight increase in the degradation behavior of the nanofibers loaded with 5FU, PLEO, and the combination of both compounds could be noticed which might be explained by the slight increase in the hydrophilicity of the blended nanofibers attributed to the hydrophilic nature of the 5FU and the variety of bioactive compounds presented in the PLEO [119].

More importantly, the slow degradation rates reported for the obtained nanofibers support their potential application to tumor tissues and ensure sustained and prolonged release and enhanced penetration of the loaded compounds within the tumor microenvironment, hence resulting in higher therapeutic efficacy of the released components against the cancer cells [89–93].

### Nanofibers release profiles

**Fig. 10:**
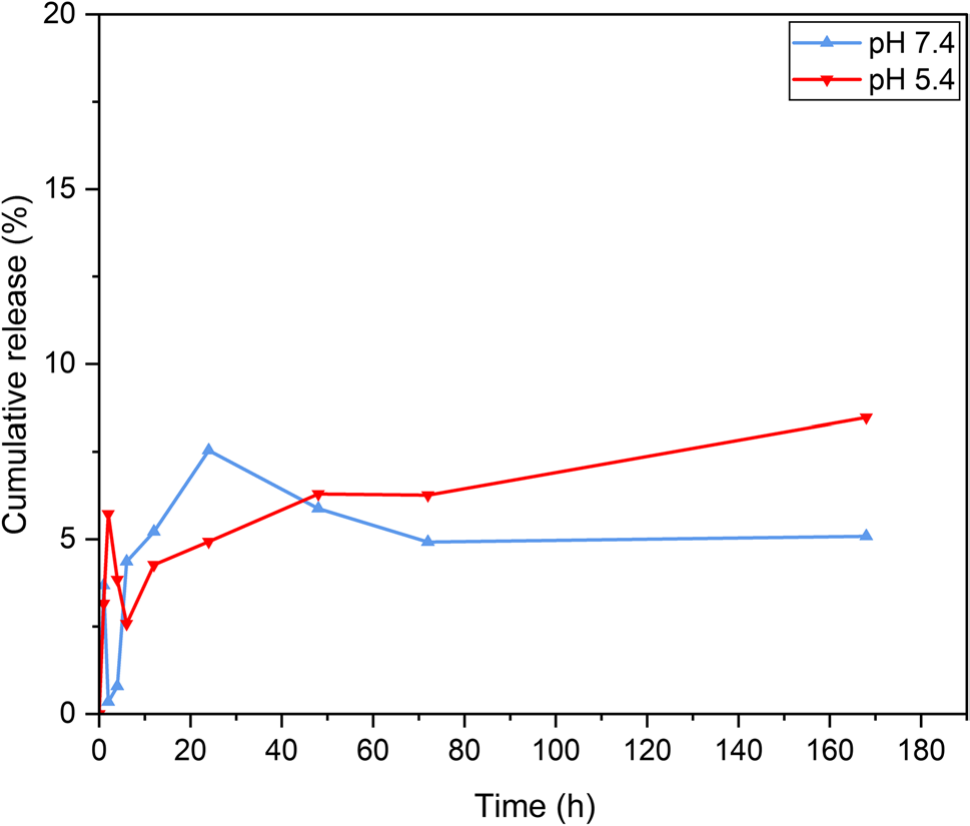
Cumulative release profile of 5FU at pH of 7.4 and 5.4

**Fig. 11:**
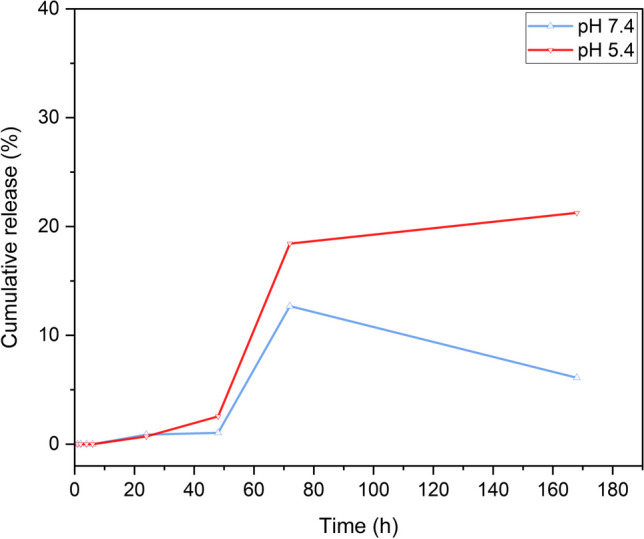
Cumulative release profile of PLEO at pH of 7.4 and 5.4

Nanofibers *in-vitro* release profiles of 5FU and PLEO are shown in Figs. 10 and 11, respectively. The release profile of the nanofibers loaded with 5FU at pH 7.4 and pH 5.4 exhibited a prolonged sustained release of 5FU over 168 h, with no evidence of burst release. As can be illustrated in Fig. 10, the released amounts of 5FU reached 4.9 ± 0.002% and 6.3 ± 0.01% at pH 7.4 and pH 5.4, respectively, over the first 72 h. Also, the released amounts of 5FU slightly increased to reach 5.1 ± 0.09% and 8.5 ± 0.03% at pH 7.4 and pH 5.4, respectively, at the end of the time interval of this study at 168 h. The higher release rates of 5FU at the acidic pH value of 5.4 indicate a promising finding that the release of the drug molecules from the nanofibers in the tumor environment is higher than their release in physiological environment (pH 7.4), demonstrating a higher targeting ability of the drug by the nanofiber and a higher safety margin exerted towards the normal physiological tissues that surround tumor microenvironment. It is worth noting that the drug was released steadily within the first few hours, and then a very slight decrease in the release happened which might refer to the presence of a few traces of the drug molecules physically adsorbed or attached near the nanofibers surface [128]. After that, the drug molecules showed a sustained release behavior.

On the other hand, the release profile of PLEO depicted a sustained prolonged release behavior over 168 h, without burst release (Fig. 11). The release rates detected at pH 7.4 and pH 5.4 were 12.7 ± 0.01% and 18.4 ± 0.03%, respectively, over the first 72 h. However, while the released amounts of PLEO dropped to 6.1 ± 0.5% at pH 7.4, the release increased to 21.3 ± 0.4% at pH 5.4, at the end of the time interval of this study at 168 h. It is worth noting that the nanofibers showed no release of PLEO within the first 6 h which might be interpreted by the efficient entrapment of the PLEO components within the nanofiber structures and the higher hydrophobicity of the PLEO when compared to the hydrophilic nature of 5FU. More importantly, the significantly higher release rates of PLEO shown at pH value of 5.4, compared to the decreased amounts released at pH 7.4, interestingly refer to the higher response and the greater targeting ability of the PLEO components towards the acidic medium of the tumor microenvironment.

Furthermore, the prolonged release profiles of both 5FU and PLEO might be explained by the slow and steady diffusion of these compounds from the nanofibers, in which the slowly biodegradable structures of the nanofiber mats provide a higher surface area and a prolonged contact time for their entrapped molecules towards their surrounding biological media. Similar observations were reported previously [93, 129].

The current findings suggest that the nanofiber mats triggered sustained prolonged release of both 5FU and PLEO. Additionally, the release of loaded compounds from the nanofibers was higher in acidic pH of tumor microenvironment, and this should undoubtedly maximize their therapeutic efficacy with lower off-target side effects.

On the other hand, different release kinetic models were developed to further investigate the release of 5FU and PLEO from the polymeric matrix of PCL NFs and to examine the regression analyses of the obtained release curves. Table 2 shows the correlation coefficient values (R^2^ ) for the 5FU-PCL-NFs and PLEO-PCL-NFs samples at two pH values.

**Table 2:** Developed release kinetic mathematical models and regression coefficients (R^2^) of 5FU-PCL-NFs and PLEO-PCL-NFs at two pH values

Sample	Zero-order	First-order	Higuchi	Korsmeyer-Peppas (n values for confirming release mechanism)	
	R^2^	R^2^	R^2^	n	Mechanism of release
5FU-PCL-NFs (pH 7.4)	0.767	0.778	0.901	0.424	Fickian diffusion
5FU-PCL-NFs (pH 5.4)	0.905	0.91	0.985	0.187	Fickian diffusion
PLEO-PCL-NFs (pH 7.4)	0.987	0.986	0.874	0.224	Fickian diffusion
PLEO-PCL-NFs (pH 5.4)	0.969	0.965	0.813	0.439	Fickian diffusion

*(n* < *0.5 signifies a Fickian diffusion; n* = *0.5 refers to a non-Fickian release; 0.5* < *n* < *1 denotes a case-II transport release)*

Interestingly, the obtained release data could fit several kinetic model equations (Higuchi, zero- and first-orders), as seen in Table 2. For 5FU-PCL-NFs, the release data could best fit the Higuchi kinetic model of release. For PLEO-PCL-NFs, the release data best fits the zero and first-order kinetic models of release. Nevertheless, the coefficient of release (n) values, obtained by fitting the results into Korsmeyer-Peppas equation, were reported with values of less than 0.5, referring to the Fickian diffusion mechanism of release governing the release of both compounds from their corresponding nanofibers. The current release data could further be associated with the sustained and significantly prolonged release of the loaded compounds, owing to the highly hydrophobic nature of the PCL nanofibers, resulting in a slow and a steady diffusion pattern of release (Fickian transport) for both compounds from the matrix of the polymeric nanofibers, verifying that the release process was mainly governed by diffusion. These findings support the advantageous properties of the current polymeric nanofiber system as a promising drug delivery system for the local and controlled release of similar bioactive compounds presented in this work. Similar observations were reported previously [98–100].

### Nanofibers antioxidant activity

A DPPH antioxidant assay was utilized to investigate the antioxidant activity of the obtained nanofibers, in which the stable free radical of DPPH**⋅** is used as a probe to measure the scavenging activity. DPPH**⋅** stable free radicals give a purple color in methanolic or ethanolic solutions. The color of the solution turns into yellow once the DPPH**⋅** free radicals scavenged by scavenger molecules (i.e., antioxidant molecules) which induce the reduction of the free radicals of DPPH**⋅** into DPPH-H. Spectrophotometrically, the purple solution gives a maximum absorption at 517 nm, and the absorbance reduces simultaneously when the color changes from purple to yellow due to the reduction of the free radicals by the antioxidant molecules. Thereafter, the antioxidant activity (DPPH scavenging ability) can be measured by the reduction in absorbance at 517 nm. [130] The antioxidant activities of the PCL-NFs and 5FU-PCL-NFs were 21.55% and 19.52%, respectively. However, the PLEO-PCL-NFs and 5FU-PLEO-PCL-NFs revealed remarkable antioxidant activities of 54.45% and 53.94%, respectively. These results are in agreement with previous reports [131–135]. The higher scavenging activity revealed with the nanofibers loaded with the PLEO is mainly ascribed to the terpenes (α-pinene and β-myrcene), oxygenated monoterpenes (myrtenol, thymol, and carveol), and other polyphenolic compounds shown in the GC–MS analysis [131, 132].

These results can significantly support the anticancer properties of the loaded nanofibers, scavenging reactive oxygen species (ROS) and free radicals. ROS are significantly increased in tumor cells, compared to normal cells, exceeding the capacity of available antioxidants [136]. Additionally, ROS serve as signal amplifiers to exaggerate the angiogenesis, invasion, migration, and proliferation of cancer cells while harming normal cellular components (i.e., lipids, nucleic acids, and proteins), ultimately leading to cell death [136]. Hence, the significant antioxidant properties of the obtained nanofibers support the promising anticancer properties of the developed nanosystem.

### Nanofibers anticancer Activity

**Fig. 12:**
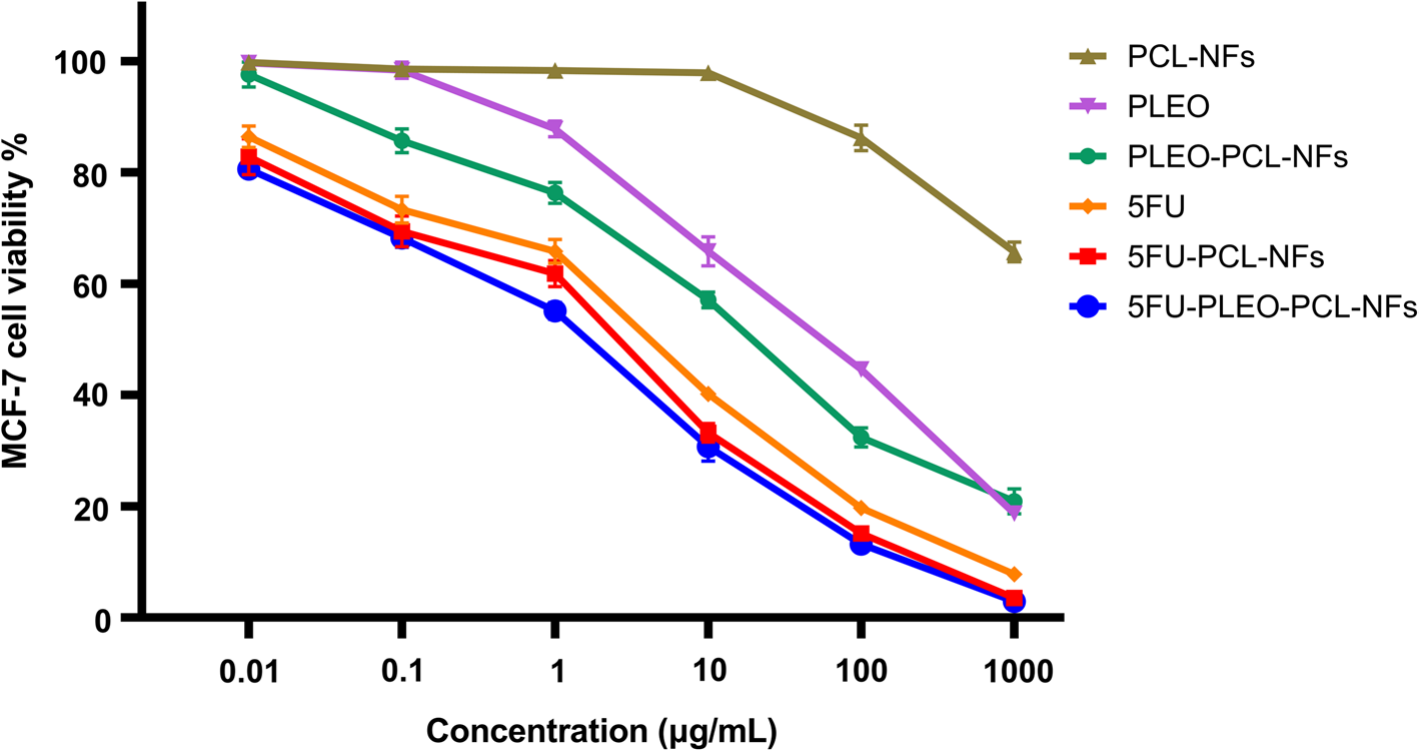
MCF-7 cancer cell viability upon treatment for 72 h with 5FU, PLEO, PCL-NFs, PLEO-PCL-NFs, 5FU-PCL-NFs, and 5FU-PLEO-PCL-NFs, using a range of concentrations equivalent to 0.01, 0.1, 1, 10, 100, and 1000 μg/mL of 5FU and/or PLEO. Data were shown as the mean of three independent experiments ± SD

**Fig. 13:**
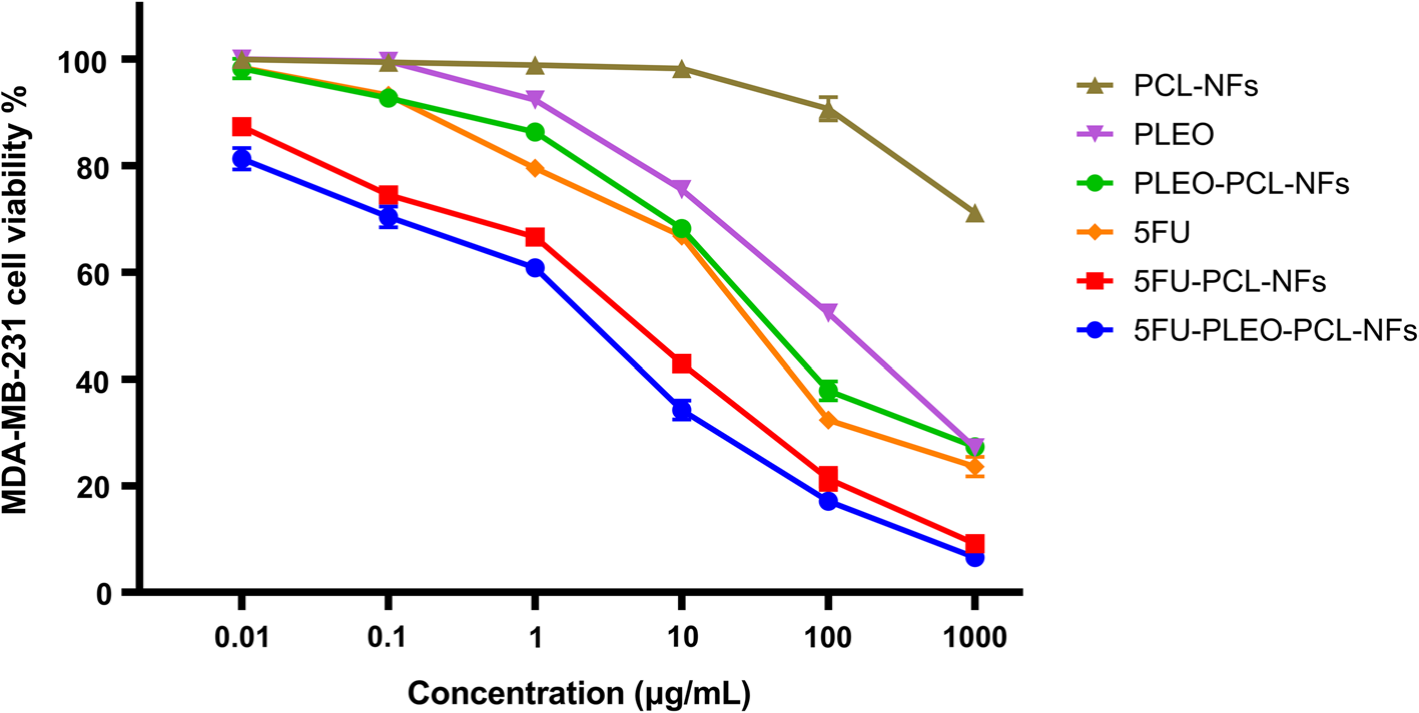
MDA-MB-231 cancer cell viability upon treatment for 72 h with 5FU, PLEO, PCL-NFs, PLEO-PCL-NFs, 5FU-PCL-NFs, and 5FU-PLEO-PCL-NFs, using a range of concentrations equivalent to 0.01, 0.1, 1, 10, 100, and 1000 μg/mL of 5FU and/or PLEO. Data were shown as the mean of three independent experiments ± SD

**Fig. 14:**
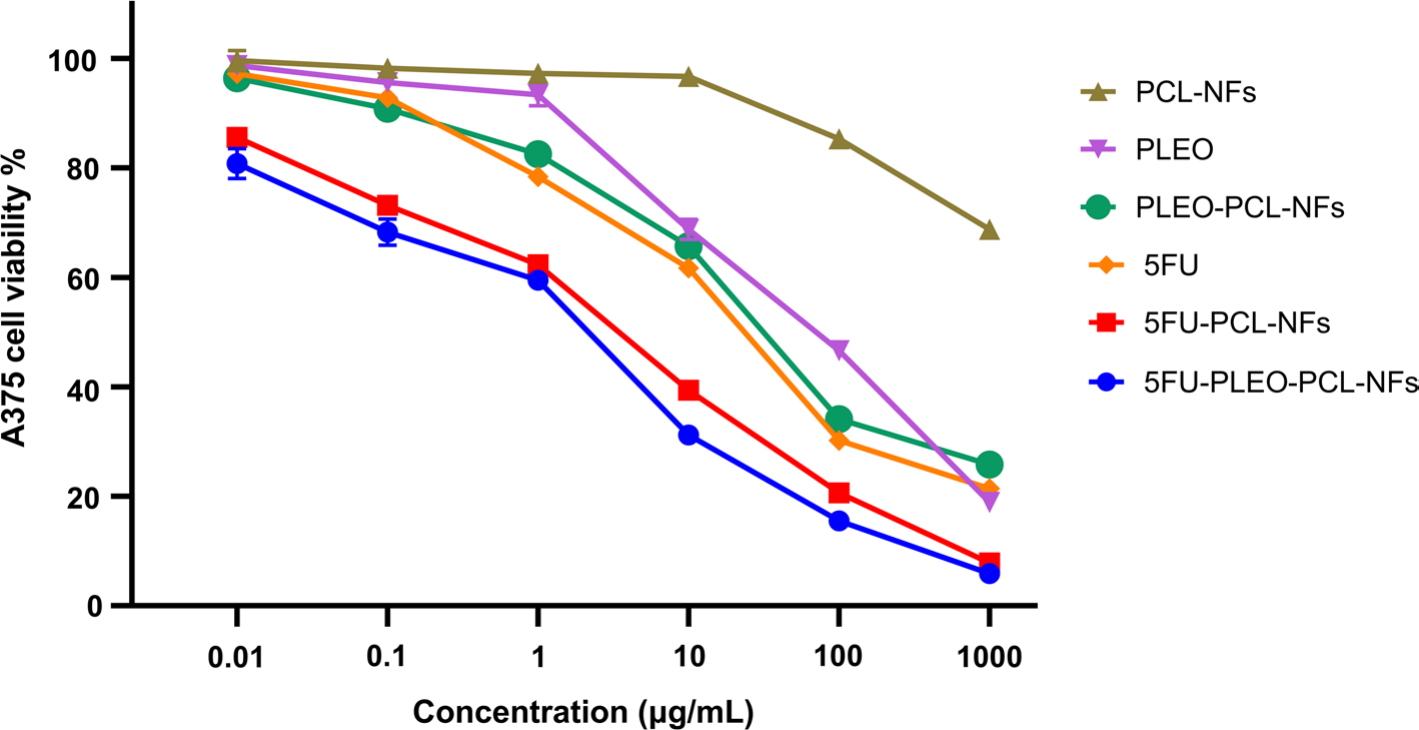
A375 cancer cell viability upon treatment for 72 h with 5FU, PLEO, PCL-NFs, PLEO-PCL-NFs, 5FU-PCL-NFs, and 5FU-PLEO-PCL-NFs, using a range of concentrations equivalent to 0.01, 0.1, 1, 10, 100, and 1000 μg/mL of 5FU and/or PLEO. Data were shown as the mean of three independent experiments ± SD

The cytotoxicity of 5FU, PLEO, and the developed nanofibers (PCL-NFs, 5FU-PCL-NFs, PLEO-PCL-NFs, and 5FU-PLEO-PCL-NFs) was assessed against MCF-7 and MDA-MB-231 human breast cancer cell lines and against A375 melanoma cancer cell line (Table 3). For this purpose, an MTT assay showing the cells viability after 72 h of treatment was performed. The cytotoxicity results expressed by IC50 values and cells viability are shown in Table 3 and Figs. 12, 13, and 14.

**Table 3:** Cytotoxic activity of 5FU, PLEO, and loaded nanofibers, using a range of concentrations equivalent to 0.01, 0.1, 1, 10, 100, and 1000 μg/mL of 5FU and/or PLEO, against MCF-7, MDA-MB-231, and A375 cell lines after 72 h of treatment

	IC_50_ on MCF-7 (μg/mL)	IC_50_ on MDA-MB-231 (μg/mL)	IC_50_ on A375 (μg/mL)
PCL-NFs	> 300^a^	> 300^a^	> 100^a^
PLEO	67.31	93.58	73.21
PLEO-PCL-NFs	12.10	17.49	15.32
5FU	7.87	16.14	12.32
5FU-PCL-NFs	5.88	9.3	7.01
5FU-PLEO-PCL-NFs	3.82	5.28	4.24

^**a**^Values preceded by the following sign ( >) indicate that that tested concentration(s) failed to show an IC50

Free PLEO showed strong cytotoxic activity against MCF-7, MDA-MB-231, and A375 cell lines with IC50 values of 67.31, 93.58, and 73.21 μg/mL, respectively. These findings support previous studies which reported the anticancer activity of PLEO against different tumor tissues such as prostate [79, 80], leukemia [69], lung [68, 137, 138], colon [67, 139–141], and ovarian cancers [142]. The anticancer activity of the PLEO was established via a series of mechanisms such as suppressing the VEGF (vascular endothelial growth factor) release, activating certain signaling pathways (*e.g.*, caspases 3, 8, 9), and downregulating the expression of several genes, proteins, and chemokines (e.g., NF-κB, RhoA, GTPases, and Ras) [67–69, 79, 80, 137–142]. Hence, these mechanisms helped in reducing the viability of cancer cells and tumor volumes, inducing apoptosis, and inhibiting cancer cells proliferation and tumor neovascularization [67–69, 79, 80, 137–142].

Moreover, PLEO-PCL-NFs showed a remarkable increase in the cytotoxic activity of PLEO by 5 to 6 folds against MCF-7, MDA-MB-231, and A375 cell lines with IC50 values of 12.10, 17.49, and 15.32 μg/mL, respectively. This enhancement might be attributed to the local and prolonged release behavior provided by the nanofibers in addition to the better protection and incorporation of the PLEO achieved by the biostable nanoformula of the PCL polymeric nanofibrous structure. The prolonged contact of the PLEO-PCL-NFs provided a sustained release and consistent diffusion of the PLEO which could subsequently increase their targeting ability and contact time with the cancerous environment, enhancing their transport, penetration, and diffusion through the membranes of the cancer cells [143].

Pure 5FU exhibited a potent anticancer activity against MCF-7, MDA-MB-231, and A375 cell lines with IC50 values of 7.87, 16.14, and 12.32 μg/mL, respectively. 5FU is an antimetabolite anticancer agent and has been widely utilized for the treatment and prevention of several sorts of cancers, including breast, colorectal, and skin cancers [144–146], where it mainly suppresses tumor cells replication via inhibiting thymidylate synthase enzyme synthesis and suppressing its metabolites incorporation into DNA and RNA [144–146].

Furthermore, the incorporation of 5FU into PCL-NFs resulted in higher cytotoxic activities against MCF-7, MDA-MB-231, and A375 cell lines with IC50 values of 5.88, 9.3, and 7.01 μg/mL, respectively. In a similar manner to what was revealed with the PLEO-PCL-NFs, the increase in the anticancer effect of 5FU-PCL-NFs can be attributed to the higher stability, protection, and prolonged and sustained release of 5FU upon loading inside PCL-NFs, increasing its therapeutic efficacy, targeting ability, and penetration properties across the tumor cells. [143]

On the other hand, 5FU-PLEO-PCL-NFs showed significant improvement in cytotoxicity against the three cell lines with the highest IC50 values when either compared to the free PLEO, pure 5FU, or their corresponding NFs (PLEO-PCL-NFs and 5FU-PCL-NFs). The IC50 values of the 5FU-PLEO-PCL-NFs against MCF-7, MDA-MB-231, and A375 were 3.82, 5.28, and 4.24 μg/mL, respectively. Based on these results, it can be noted that the 5FU-PLEO-PCL-NFs showed a synergistic anticancer activity against all the cell lines treated, in which the presented viabilities of the cancer cells treated with 5FU-PLEO-PCL-NFs were significantly lower than treatments with other nanofibers (i.e., with PLEO-PCL-NFs or 5FU-PCL-NFs).

These findings support the local application of the developed nanofibers loaded with PLEO and 5FU drug for the treatment and/or post-surgical prevention of breast and melanoma skin cancers. More importantly, despite the results showing a proper synergistic effect of both compounds when loaded concomitantly in nanofibers, the use of a natural product with an anticancer activity is promisingly preferred to subside the well-known systemic and major detrimental side effects associated with chemotherapeutic administration.

In addition to the above-mentioned possible mechanisms of action of PLEO and 5FU against tumor tissues, including breast and skin cancers, the antioxidant properties of the PLEO loaded nanofibers can also present an additional possible interpretation of the augmented anticancer activities, scavenging free radicals and ROS generated by cancer cells [136]. Also, the major component of the extracted PLEO, α-pinene coupled with other monoterpenes induce apoptosis and cancer cell-cycle arrest (G2/M) in ovarian cancer and hepatocellular carcinoma [103–108]. Additionally, α-pinene was reported to suppress cancer invasion by inhibiting matrix metalloproteinase-9 expression in breast cancer [109] and activate natural killer cells (lymphocytes), increasing their cytotoxicity against colon cancer (CT-26) [110]. Moreover, PLEO was shown to induce apoptosis against ovarian (SKOV-3) and breast cancer cells (MCF-7), mainly targeting the cell-cycle sub-G1 phase [41].

**Fig. 15:**
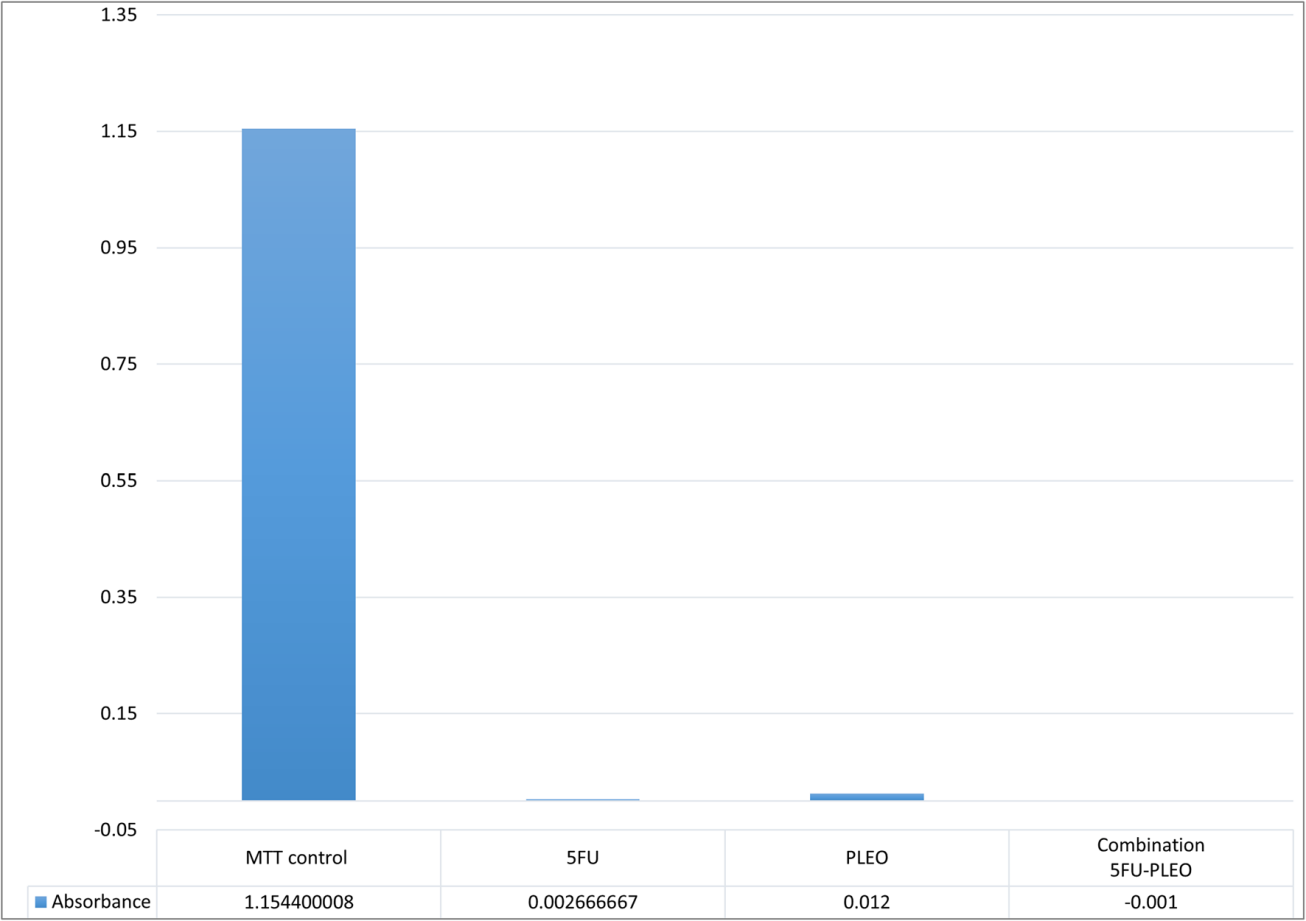
Absorbance values (at 570 nm) of the MTT solution added to free 5FU, PLEO, and their combination (5FU-PLEO), compared to an MTT positive control (MTT added to cultured HSF cells)

On the other hand, none of the MTT interference tests performed on free 5FU, PLEO, and their combination revealed any interference that might influence the MTT assay results since the obtained absorbance values were negligible compared to the MTT positive control (MTT added to cultured HSF cells) (Fig. 15) which eliminated the possibility of MTT false positive results.

Finally, despite some studies observed a biphasic concentration–response effect when testing phytochemicals on cultured cancer cells [147], no such behavior was observed with PLEO in this work.

## Conclusions and future perspectives

One of the most common cancer treatments is the administration of chemotherapeutics which cause severe and chronic toxicities and chemoresistance. Many natural compounds derived from various plant extracts have exerted potent anticancer activities. Particularly, EOs have been established with great potential for various preventive and therapeutic strategies amongst different tumors. Various tumorous tissues have shown great recession after being targeted and treated with EOs of different plants. Several malignancies were targeted by EOs, such as leukemia, hepatoma, breast tumors, gastric malignancies, glioma, and pulmonary and colorectal cancers. EOs of *P. lentiscus* (PLEO) have demonstrated promising antimicrobial, antioxidant, anticancer, and anti-inflammatory properties. However, their poor solubility, bioavailability, and stability have limited their therapeutic beneficence. Hence, PLEO were encapsulated into nanofibers (NFs) to enhance their physicochemical properties and therapeutic potential and to reduce their drawbacks. PLEO and 5FU were successfully electrospun into PCL-NFs separately and in combination. Several characterization tests were performed to investigate the developed NFs. FE-SEM showed an average diameter of the NFs between 290.71 nm to 680.95 nm. FT-IR and XRD confirmed the encapsulation of both compounds and investigated the chemical structures changes among all NFs. Also, TGA analysis revealed excellent thermal stability of the NFs over a wide range of temperatures, compared to free PLEO and 5FU. Furthermore, the NFs physical integrity and mechanical strength suggested high stability of all NFs. NFs degradability study showed remarkable stability and negligible degradation rates (~ 5%) after 6 weeks, supporting their local and prolonged therapeutic uses. Additionally, the release studies showed sustainable and prolonged release profiles of PLEO (6.1%) and 5FU (5.1%), without initial burst release, at pH 7.4, over 168 h. More importantly, higher amounts were released over the same period reaching 21.3% for PLEO and 8.5% for 5FU at the acidic tumor environment (pH 5.4), indicating a promising release sustainability and a higher targeting ability of the NFs towards tumor cells. Also, a remarkable antioxidant activity of PLEO-NFs was reported with 54.45% inhibition of free radicals, using DPPH assay. In addition, NFs were revealed with promising anticancer activity against breast cancer (MCF-7 and MDA-MB-231) and melanoma cells (A375). Free PLEO showed IC50s of 67.31, 93.58, and 73.21 μg/mL against MCF-7, MDA-MB-231, and A375, respectively. PLEO-PCL-NFs depicted higher cytotoxic activities by 5-fold with IC50s of 12.10, 17.49, and 15.32 μg/mL, respectively. 5FU-PCL-NFs showed greater cytotoxic activities with IC50 values of 5.88, 9.3, and 7.01 μg/mL, respectively. Nevertheless, in case of the nanofibers loaded with both 5FU and PLEO, remarkable cytotoxic activity could be revealed with IC50s of 3.82, 5.28, and 4.24 μg/mL, respectively. Hence, NFs loaded with either PLEO or 5FU depicted greater anticancer activities in comparison to their corresponding free compounds, and even higher anticancer activities could be observed with their co-loaded NFs. Nevertheless, the safety profile was evident by testing PLEO against normal breast epithelial cell line (MCF10A) where no cytotoxicity was observed. Also, unloaded PCL-NFs showed no toxicity against cells tested in this study. These findings indicate greater cytotoxic activities of 5FU and PLEO exerted upon their loading into NFs and their possible synergistic activity showing higher anticancer activity when combined.

Loading PLEO and 5FU in biodegradable and biocompatible polymeric PCL NFs improved their stability profiles, release sustainability, and anticancer activity against breast and skin cancers. Also, PLEO loaded NFs depicted excellent antioxidant properties. More importantly, the study indicated a possible synergistic anticancer effect of the PLEO and 5FU co-loaded in PCL-NFs against breast and melanoma cancer cells. Therefore, encapsulation of natural extracts alongside chemotherapeutics in different nanoformulations would represent an effective strategy to reduce therapeutic doses and consequently side effects of chemotherapeutics. The developed PCL-NFs, co-loaded with 5FU and PLEO, present a superior biocompatible and biodegradable drug delivery system for treating melanoma and breast cancer or preventing their local recurrence following surgery.

Future studies may explore the detailed mechanisms which underly the possible synergistic effect shown with the PLEO and 5FU co-loaded NFs. Also, in vivo studies are needed to confirm the therapeutic efficacies of the developed NFs. Moreover, other chemotherapeutic agents might be loaded in similar nanoformulas to investigate the potential synergistic effects of PLEO in increasing their anticancer activity, and to lower the concentrations of chemotherapeutics and eventually minimize their systemic side effects.

### Supplementary Information

**Additional file 1:** Electrospinning conditions for the trials conducted before reaching optimal parameters for the electrospun nanofibers; and FE-SEM images for the nanofibers obtained using a different solvent system.

## Data Availability

The datasets generated during this study are available on reasonable request.
