# Supplementary material for: Synergistic anticancer effect of Pistacia lentiscus essential oils and 5-Fluorouracil co-loaded onto biodegradable nanofibers against melanoma and breast cancer
Source: Discov Nano. 2024 Feb 14;19(1):27. doi: 10.1186/s11671-024-03962-5 (PMC10866856; doi:10.1186/s11671-024-03962-5)
Supplement: Supplementary file 1 — Additional file 1: Electrospinning conditions for the trials conducted before reaching optimal parameters for the electrospun nanofibers; and FE-SEM images for the nanofibers obtained using a different solvent system. [file 11671_2024_3962_MOESM1_ESM.docx]

**Synergistic Anticancer Effect of *Pistacia lentiscus* Essential Oils and 5-Fluorouracil Co-loaded onto Biodegradable Nanofibers against Melanoma and Breast Cancer**

**Obaydah Abd Alkader** **Alabrahim *^a^*** and **Hassan Mohamed El-Said Azzazy *^ab^****

***^a^*** Department of Chemistry, School of Sciences & Engineering, The American University in Cairo, New Cairo 11835, Egypt. [Obaydah.alabrahim@aucegypt.edu](mailto:Obaydah.alabrahim@aucegypt.edu); [hazzazy@aucegypt.edu](mailto:hazzazy@aucegypt.edu).

***^b^*** Department of Nanobiophotonics, Leibniz Institute of Photonic Technology, Jena, Germany

**Corresponding authors:**

**Prof.** Hassan M. E. Azzazy

School of Sciences & Engineering

The American University in Cairo

AUC Avenue, SSE # 1184, P.O. Box 74

New Cairo, Egypt 11835

E-mail: [hazzazy@aucegypt.edu](mailto:hazzazy@aucegypt.edu)

Office: +2 02 2615 2559 (GMT+2 hr)

**Supplementary Table 1.**

**Table S1:** Electrospinning conditions for the trials conducted.

| **Electrospinning parameters** | **Conditions** | **Observations** |
| --- | --- | --- |
| **Flow rate*** | > 4 mL/h | ↑ Dripping |
|  | <4 mL/h | Taylor cone failed to develop |
| **Voltage*** | >20 KV | Instable Taylor cone |
|  | <20 KV | ↑ Dripping |
| **Solvent system** | Chloroform: Ethanol (70:30, v/v)** | Beads** (which might be explained by the insolubility of PCL polymer in ethanol.) |
|  | Chloroform: Methanol (70:30, v/v)*** | Similar findings to the presented study |

*****Flow rates and voltages reported here are associated with the solvent system used in this study (Chloroform: Methanol) (50:50, v/v).

******FE-SEM images for the NFs obtained with this solvent system are revealed below (Figures **1**, **2**, **3**, and **4** in **supplementary 2**, **3**, **4**, and **5**, respectively). It is worth noting that the Chloroform: Ethanol system was chosen at the beginning due to the polar nature of the 5FU and its solubility in Ethanol.

*******Although 5FU has a poor solubility in Methanol, the high temperature (60 °C) applied to the solvent system containing 5FU helped in dissolving the 5FU in the solvent system reported.

**Supplementary Figure 1.**


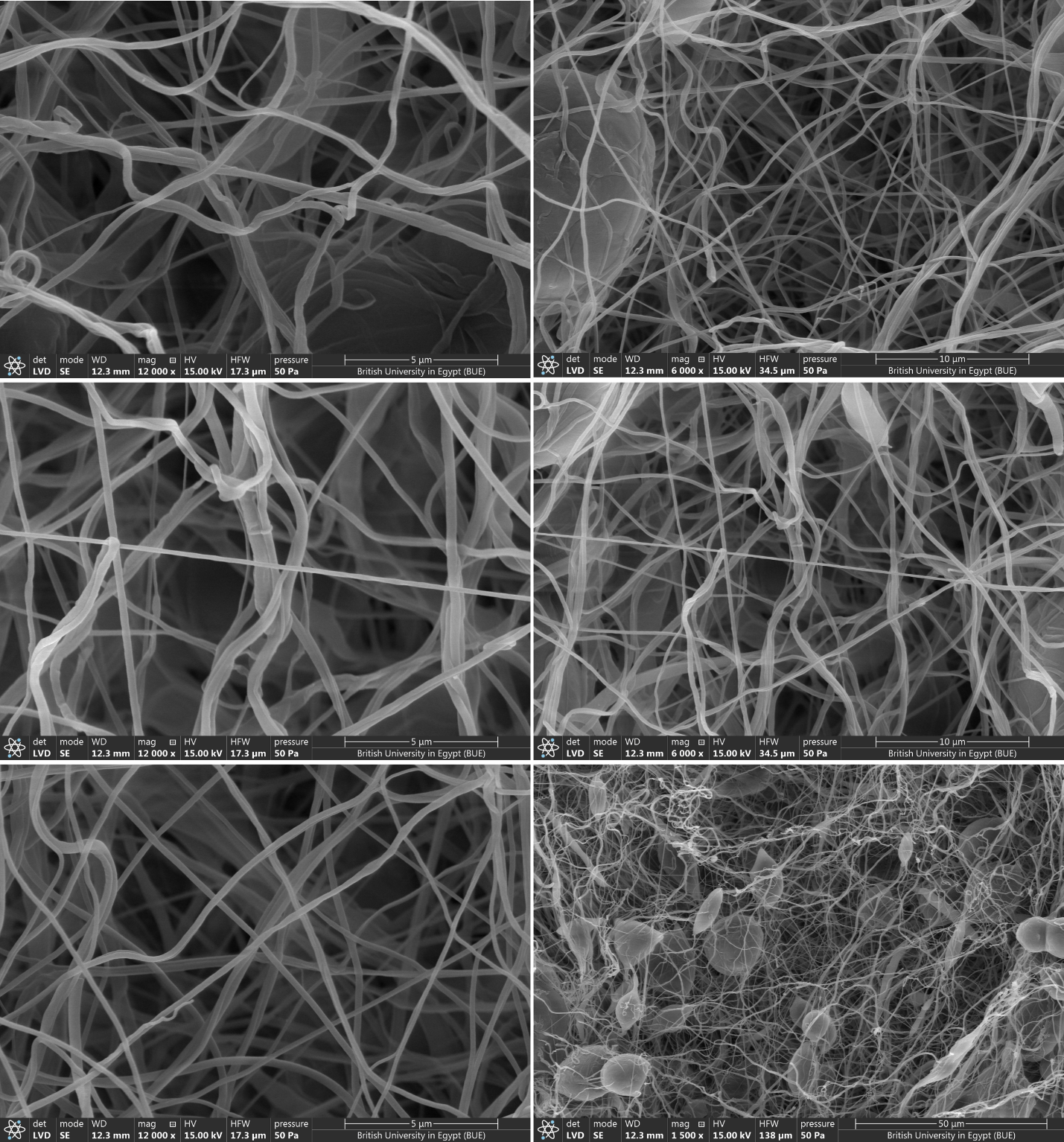


**Figure S1.** PCL-NFs prepared in a solvent system of Chloroform: Ethanol (70:30, v/v). Electrospinning parameters were 8 to 12 mL/h flow rate and 20 KV.

**Supplementary Figure 2.**


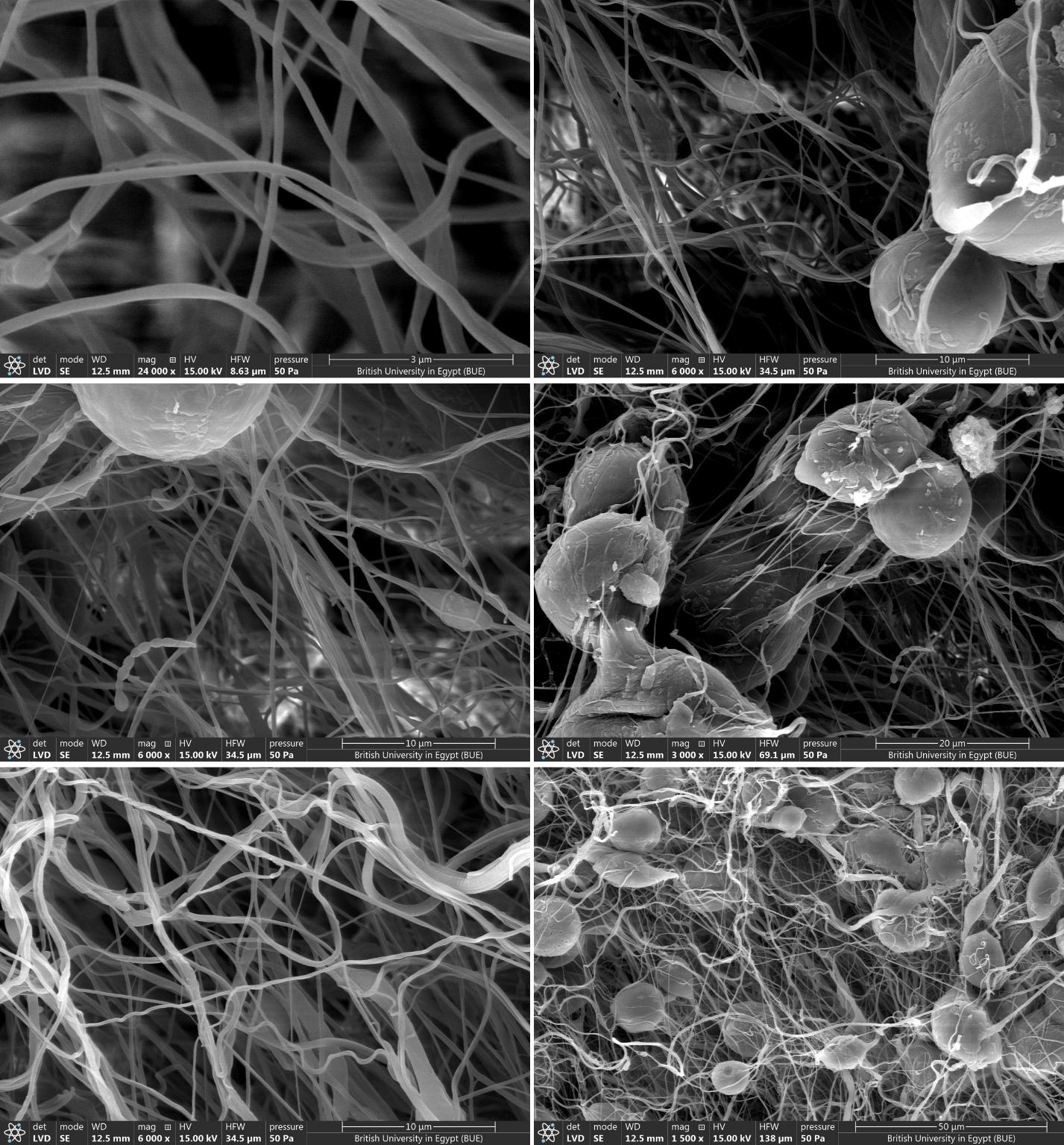


**Figure S2.** 5FU-PCL-NFs prepared in a solvent system of Chloroform: Ethanol (70:30, v/v). Electrospinning parameters were 7 mL/h flow rate and 20 KV.

**Supplementary Figure 3.**


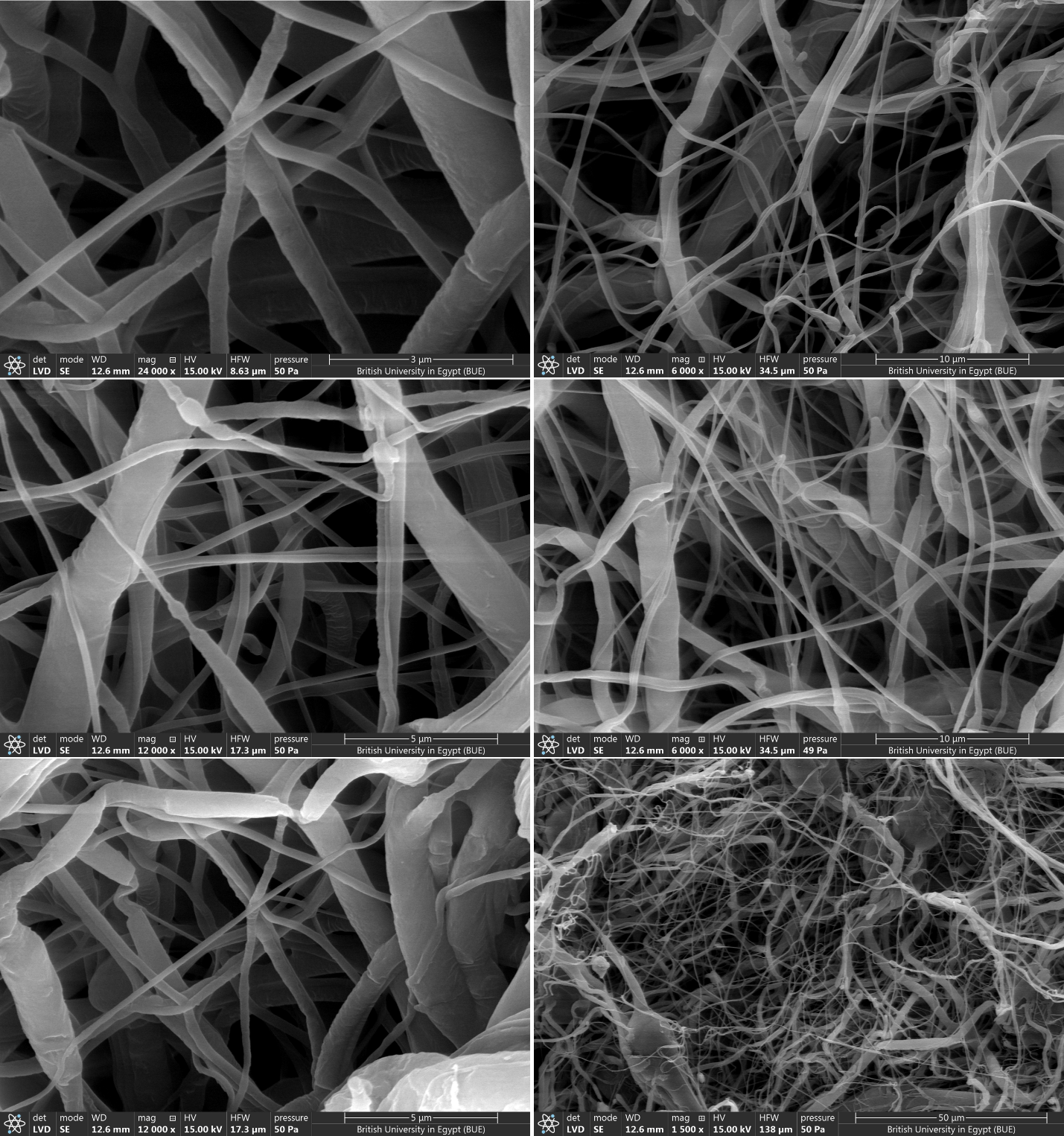


**Figure S3.** PLEO-PCL-NFs prepared in a solvent system of Chloroform: Ethanol (70:30, v/v). Electrospinning parameters were 8 mL/h flow rate and 20 KV.

**Supplementary Figure 4.**


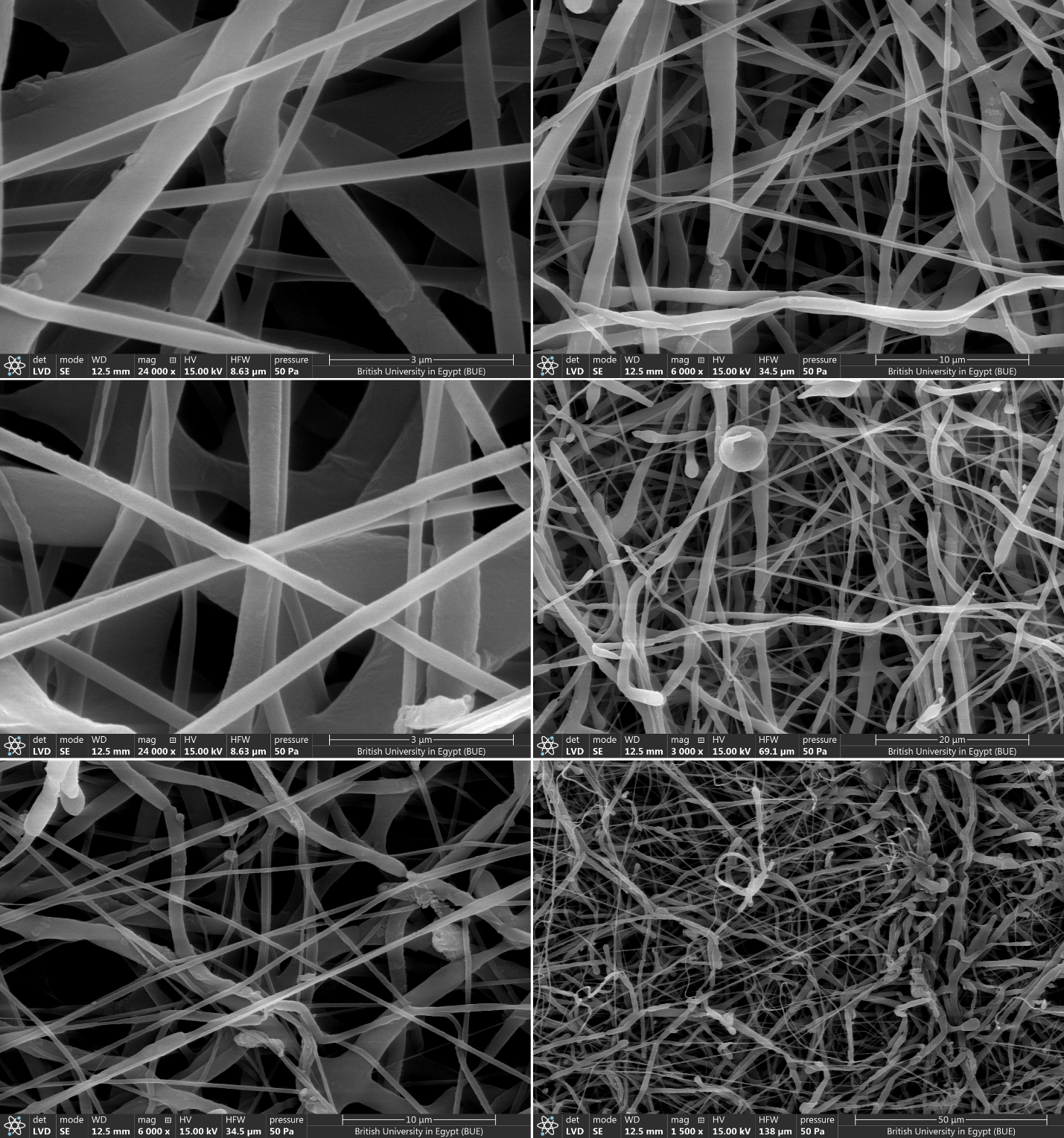


**Figure S4.** 5FU-PLEO-PCL-NFs prepared in a solvent system of Chloroform: Ethanol (70:30, v/v). Electrospinning parameters were 6 mL/h flow rate and 20 KV.
